# High-resolution Structural and Thermodynamic Analysis of Extreme Stabilization of Human Procarboxypeptidase by Computational Protein Design

**DOI:** 10.1016/j.jmb.2006.11.080

**Published:** 2007-03-02

**Authors:** Gautam Dantas, Colin Corrent, Steve L. Reichow, James J. Havranek, Ziad M. Eletr, Nancy G. Isern, Brian Kuhlman, Gabriele Varani, Ethan A. Merritt, David Baker

**Affiliations:** 1 Department of Biochemistry, University of Washington, Seattle, WA 98195, USA; 2 Department of Chemistry, University of Washington, Seattle, WA 98195, USA; 3 Howard Hughes Medical Institute, University of Washington, Seattle, WA 98195, USA; 4 Department of Biochemistry and Biophysics, University of North Carolina, Chapel Hill, NC 27599, USA; 5 EMSL High Field Magnetic Resonance Facility, PNNL, Richland, WA 99352, USA

**Keywords:** HSQC, heteronuclear single-quantum coherence, NOE, nuclear Overhauser effect, NOESY, NOE spectroscopy, RMSD, root-mean-square deviation, RDF, radial distribution function, Computational protein design, Rosetta, Thermodynamic stabilization, High-resolution protein structure, Procarboxypeptidase A2

## Abstract

Recent efforts to design *de novo* or redesign the sequence and structure of proteins using computational techniques have met with significant success. Most, if not all, of these computational methodologies attempt to model atomic-level interactions, and hence high-resolution structural characterization of the designed proteins is critical for evaluating the atomic-level accuracy of the underlying design force-fields. We previously used our computational protein design protocol RosettaDesign to completely redesign the sequence of the activation domain of human procarboxypeptidase A2. With 68% of the wild-type sequence changed, the designed protein, AYEdesign, is over 10 kcal/mol more stable than the wild-type protein. Here, we describe the high-resolution crystal structure and solution NMR structure of AYEdesign, which show that the experimentally determined backbone and side-chains conformations are effectively superimposable with the computational model at atomic resolution. To isolate the origins of the remarkable stabilization, we have designed and characterized a new series of procarboxypeptidase mutants that gain significant thermodynamic stability with a minimal number of mutations; one mutant gains more than 5 kcal/mol of stability over the wild-type protein with only four amino acid changes. We explore the relationship between force-field smoothing and conformational sampling by comparing the experimentally determined free energies of the overall design and these focused subsets of mutations to those predicted using modified force-fields, and both fixed and flexible backbone sampling protocols.

## Introduction

Natural proteins perform a startling diversity of biological functions, but comprise a miniscule fraction of the theoretical sequence–structure space that polypeptides might occupy.^1–4^ The goal of protein design is to identify new free-energy minima in this sequence–structure landscape so as to expand the functional repertoire of polypeptides beyond that observed in nature.^5–9^ The design of new proteins should allow for the creation of novel molecular machines and therapeutics but requires an accurate description of the forces that govern protein structure and folding.^10,11^ The last decade has witnessed tremendous advances in the development of *in silico* protein sequence and structure optimization algorithms. They have been applied successfully to completely redesign^12^ and thermodynamically stabilize natural protein folds,^13^ to create novel^14^ and thermodynamically-stabilized enzymes,^15^ to redesign protein–protein^16^ and protein–ligand^17^ interactions, and to create extremely stable novel protein structures.^18,19^ While structural validation in a few cases has confirmed the high-resolution accuracy of the designs,^12,15,16,18–20^ the total repertoire of high-resolution structures of computationally designed proteins remains small. Structural characterization of designed proteins is essential for validation of the design model and for evaluating the accuracy of the underlying force-fields.

In a large-scale evaluation of our computational protein design methodology, we used RosettaDesign to completely redesign the sequence of nine small globular proteins.^13^ The redesign of the activation domain of human procarboxypeptidase A2, AYEdesign, was the most successful redesign; it had a native-like secondary structure profile, was rigid and well folded, was stabilized dramatically over its wild-type counterpart,^13^ and folded much faster and unfolded much slower than the wild-type protein.^21^ We have now determined high-resolution crystal and NMR structures of AYEdesign, to evaluate the atomic-level accuracy of the RosettaDesign protocol. We use the information gleaned from these structural studies to design and characterize a new series of AYE mutants that gain significant thermodynamic stability with a minimal number of mutations. The analysis of these results provides insight into the coupling between force-field smoothing and the extent of conformational sampling in high-resolution protein modeling and design.

## Results and Discussion

RosettaDesign was previously used to redesign completely the sequence of the activation domain of human procarboxypeptidase A2.^13^ The 1.8 Å crystal structure of the wild-type protein (1AYE)^22^ was used as a template for the design simulation, allowing all amino acids except cysteine at all 70 positions. The final sequence chosen for experimental study, AYEdesign, differed from the wild-type protein by 68% over all residues and 33% over core residues. Far-UV circular dichroism (CD) spectroscopy, 1D ^1^H nuclear magnetic resonance (NMR) spectroscopy, and chemical and thermal denaturation experiments showed that AYEdesign adopted a well-folded, rigid structure, with a secondary structure profile very similar to that of the wild-type protein. AYEdesign was found to be extremely stable; its folded structure is impervious to boiling and it is greater than 10 kcal/mol more stable than the wild-type protein (Table 2). It also folds ∼1000-fold faster and unfolds ∼20,000-fold slower than the wild-type protein.^21^ To extend the comparison between AYEdesign and its wild-type parent to atomic resolution, we have now determined both crystal and solution NMR structures of AYEdesign.

We produced and crystallized a selenomethionyl (SeMet)-substituted variant of AYEdesign (AYEdes_VJQ), and solved the x-ray crystal structure of AYEdes_VJQ to a resolution of 2.1 Å by direct rebuilding into an unbiased multiple-wavelength anomalous dispersion (MAD) electron density map and residual difference Fourier maps. The final *R*_work_ and *R*_free_ were 0.20 and 0.27, respectively (Supplementary Data Table S1). The asymmetric unit of the crystal contains two independent protein chains. The N-terminal 70 residues of each chain exhibit the expected procarboxypeptidase fold of the parent 1AYE design target. The C^α^ RMSD from the computational model is 1.68 Å and 1.28 Å for chain A and B, respectively, and this improves to 1.13 Å and 0.65 Å when 66 of the 70 residues are considered for chain A and B, respectively (Figure 2(a)).

**Supplementary Data Figure S1:**
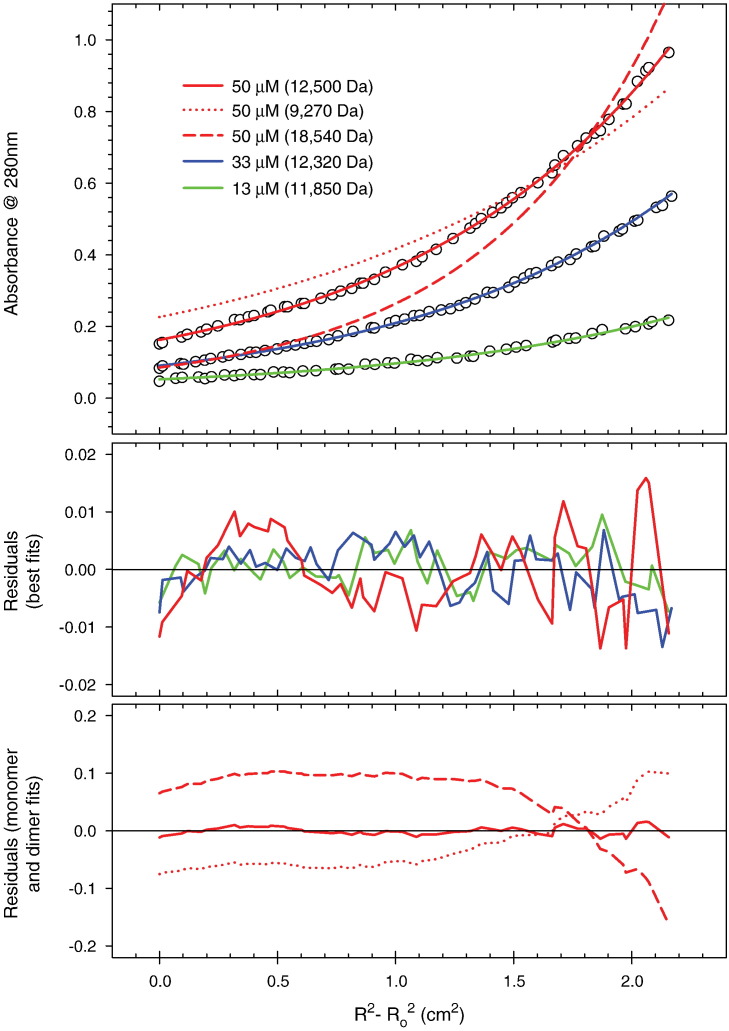


**Figure 1:**
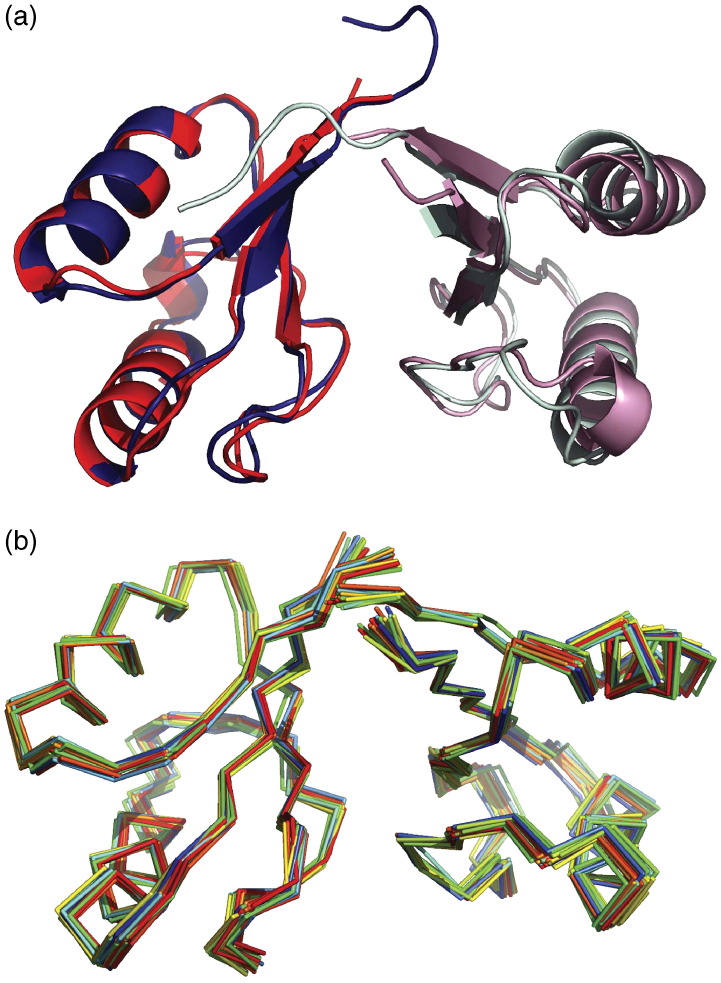
AYEdesign X-ray and NMR structures. (a) The AYEdesign X-ray crystal structure (AYEdes_VJQ, chain A in light blue, chain B in dark blue) and NMR solution structure (AYEdes, chain A in pink, chain B in red) are superimposed and shown as ribbons. The protein forms a symmetric dimer that buries 740 Å of surface area of the back-face of the β-sheet, with a gap volume index of 2.37; these values are close to the average values observed for heterodimer interactions, but indicate a weaker interaction than is typical for homodimers or permanent protein complexes.^52^ (b) The top 20 NMR models from the final AYEdes structure calculation are shown as C^α^ backbones (different color for each model). The ensemble pair-wise RMSD is 0.57(±0.18) Å over backbone atoms and 1.09(±0.11) Å over heavy-atoms in residues 3–71 in both subunits.

Two AYEdes_VJQ monomers associate to form a dimer in the crystal. Dimerization is mediated, in part, by the C termini of the two chains, which form an anti-parallel β-sheet consisting of residues 66–73 from chain A and residues 64–70 from chain B (Figure 1(a)). However, the contribution of chain A to the β-sheet includes three residues from the cleavable linker sequence that are not part of the designed sequence. Gel-filtration chromatography studies (data not shown) of the AYEdes_VJQ construct showed that the protein exists predominantly as a dimer at 10–100 μM (where thermodynamic properties of the original AYEdesign construct were measured), as well as at crystallography concentrations (≥1 mM). Since the dimer in the crystal structure was mediated at least partly by extra C-terminal residues not considered in the original design, we prepared a new construct, AYEdes, with an N-terminal His_6_ tag followed only by the 70 designed residues of AYEdesign. Consistent with our original biophysical characterization, the AYEdes protein exists predominantly as a monomer at concentrations of 10–100 μM as judged by chromatography, but exhibits partial dimeric character at higher concentrations (≥1 mM) (data not shown). Analytical ultracentrifugation suggests that AYEdes exists in a monomer–dimer equilibrium with an estimated *K*_d_ of ∼150 μM (Supplementary Data Figure S1). This weak association implies that protein dimerization plays an insignificant role in the extreme thermodynamic stabilization of the designed monomer, and that the dimer dissociates well before the monomer unfolds. We confirmed this assumption by repeating CD equilibrium denaturation experiments at multiple concentrations of AYEdes, where we observe that the melting curves and unfolding transitions for 5 μM, 50 μM, and 100 μM protein are entirely coincident. This result allowed us to fit the observed two-state unfolding of AYEdes as equilibrium denaturation between folded monomers and unfolded monomers.

**Supplementary Data Figure S2:**
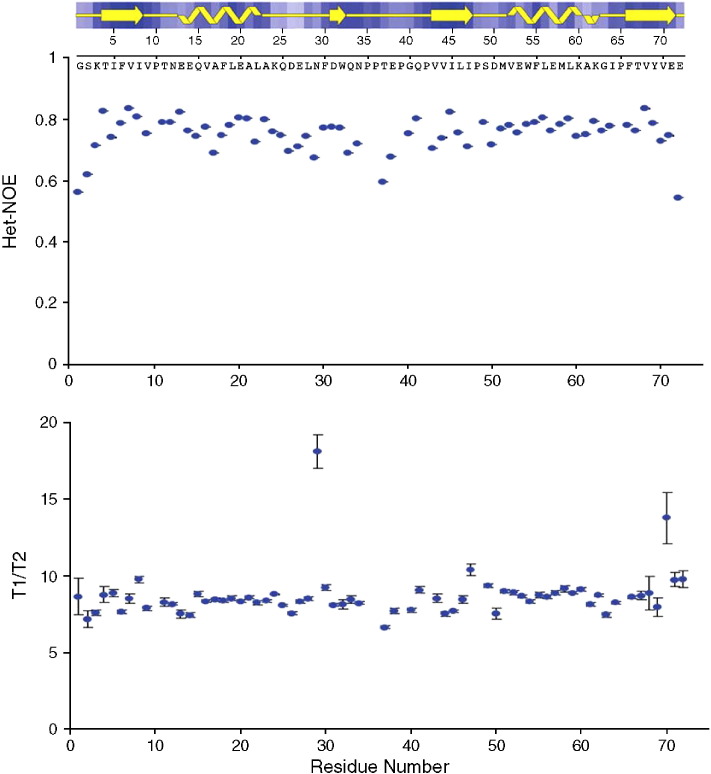


**Figure 2:**
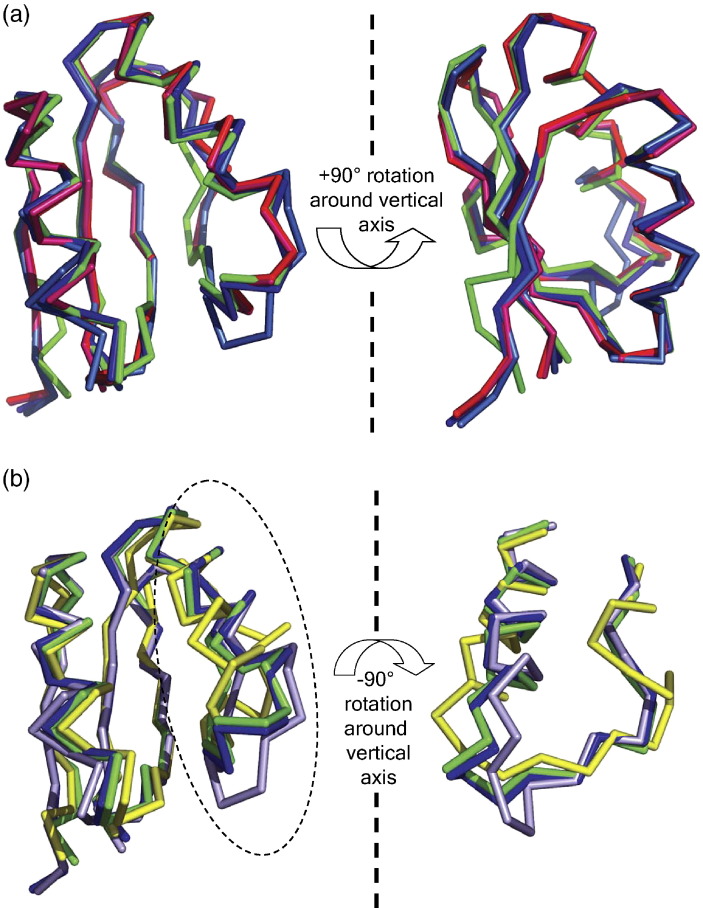
Comparison of AYEdesign computational model and experimentally determined structures. (a) Both chains of AYEdes_VJQ (light and dark blue) and AYEdes (pink and red) are superimposed on the AYEdesign computational model (green), and are shown as C^α^ backbones in two orientations (related by a +90° rotation around the vertical axis in the plane of the page). The C^α^ RMSD from the computational model is 1.68 Å, 1.28 Å, 1.51 Å, and 1.51 Å for chain A and B of AYEdes_VJQ and AYEdes, respectively, and this improves to 1.13 Å, 0.65 Å, 1.05 Å and 1.05 Å, respectively, when 66 of the 70 residues are considered. (b) The two chains in AYEdes_VJQ (light and dark blue) differ notably from each other in the conformation of the loop containing residues 25–27. The backbone of AYEdes_VJQ chain B in this region is effectively superimposable with the AYEdesign computational model (green) as well as with the two chains of the AYEdes NMR structure, but the backbone of AYEdes_VJQ chain A deviates at this point. Interestingly, this corresponds to one of two points at which an insertion or deletion distinguishes the sequence families of procarboxypeptidase A (the template in this study) and procarboxypeptidase B; residues 25 and 26 are deleted from the procarboxypeptidase B sequence. The AYEdes_VJQ chain A backbone in the present structure does not, however, adopt the conformation of procarboxypeptidase B observed in PDB entry 1KWM (yellow), where the entire α-helix equivalent to residues 11–24 in the current sequence is displaced to one side.

To assess whether removal of the extra C-terminal tag residues had any impact on the atomic-level structure of AYEdesign, we determined the NMR solution structure of the AYEdes construct. The 1D ^1^H spectra and 2D ^1^H-^15^N heteronuclear single-quantum coherence (HSQC) spectra of AYEdes exhibit the features of a well-folded protein (Figure 4), with well-dispersed NH resonances of uniform intensity.^13^ Protein backbone and side-chain assignments were obtained by standard procedures, as described in Materials and Methods. The uniform ^1^H-^15^N heteronuclear nuclear Overhauser effect (NOE) values (∼0.75) recorded for AYEdes indicate a conformationally rigid fold in solution reflecting the observed thermodynamic stability of this protein (Supplementary Data Figure S2). The *T*_1_/*T*_2_ ratios measured for AYEdes give a correlation time of 10.64 ns, which is consistent with homodimeric association under the conditions used for NMR.^23^ Notably, the HSQC spectrum contains a single set of cross-peaks for each NH in the protein, indicating a fully symmetric association in solution. Structure determination was conducted in a two-step process; a partly-automated iterative step dominated by NOE-derived distance constraints for generating models of a single subunit of AYEdes, followed by a second refinement step for building the symmetric homodimer model using interfacial NOE constraints obtained from 3D ^12^C-edited-^13^C-filtered NOE spectroscopy (NOESY) data. In the final calculation, 100 structures (chains A and B) were generated from the random-coil conformation. The 20 lowest energy structures (Figure 1(b)) had an average Cyana^24^ target function of 2.75(±0.10) Å^2^ and an ensemble pair-wise root-mean-square deviation (RMSD) of 0.57(±0.18) Å over backbone atoms and 1.09(±0.11) Å over heavy-atoms in residues 3–71 in both subunits (Supplementary Data Table S2). There was no distance constraint violated by more than 0.2 Å, and no angle constraint violated by more than 1.5°. When the ensemble was analysed with PROCHECKNMR,^25^ all dihedral angles were found in the allowed regions of the Ramachandran plot (Supplementary Table S2). The C^α^ RMSD of the lowest energy AYEdes NMR model from the parent 1AYE crystal structure is 1.51 Å over the 70 designed residues, and improves to 1.05 Å when only the first 66 of the 70 residues are considered (Figure 2(a)). The relative rigid-body orientation of the two chains in the NMR structure is virtually identical with the AYEdes_VJQ crystal structure (Figure 1(a)). These combined structural results, together with gel-filtration analysis, suggest that at high concentrations, AYEdesign self-associates and buries the surface-exposed hydrophobic residues on the β-sheet surface, and the strand-swapping in the C-terminal tag residues (in AYEdes_VJQ) serves to strengthen the dimeric interaction.

**Figure 3:**
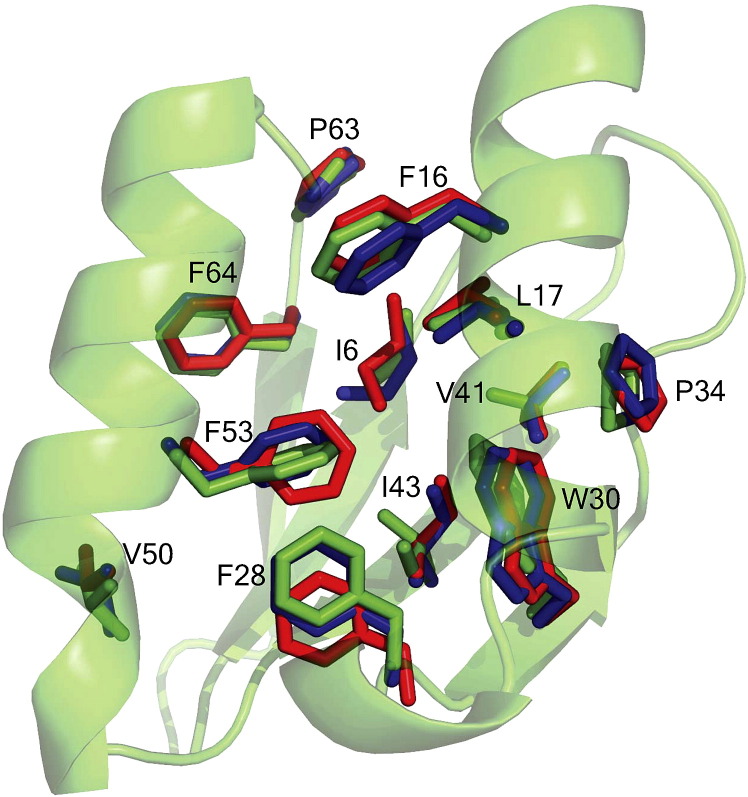
Atomic-level recovery of designed side-chain conformations in AYEdesign. The side-chains in the protein core of the AYEdesign X-ray crystal structure (AYEdes_VJQ chain B, blue) and NMR solution structure (AYEdes chain B, red) are effectively superimposable on the computational model (green). Selected side-chains are shown as sticks and the protein backbone of the computational model is shown as cartoon ribbons.

**Figure 4:**
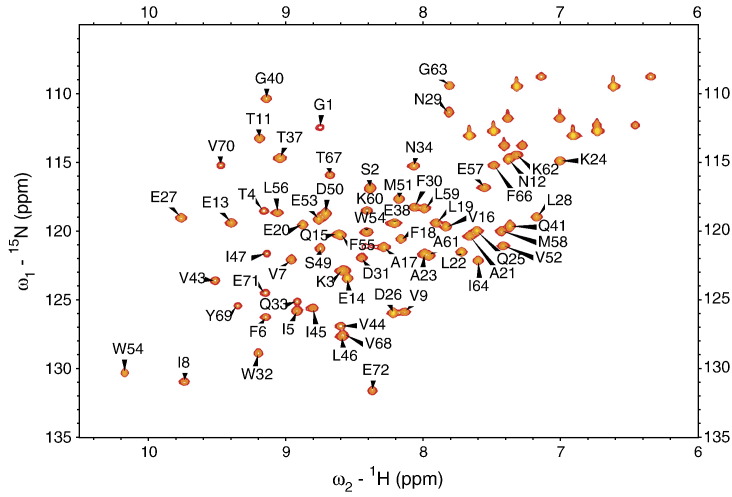
^1^H-^15^N HSQC spectrum of AYEdes. The HSQC spectrum of ∼1 mM ^15^N-AYEdes in 50 mM potassium phosphate (pH 7.0), 100 mM KCl, recorded at 298 K and 750 MHz. Peaks are labeled with the one-letter amino acid code and sequence number, unlabeled peaks in the upper right corner of the spectrum correspond to side-chain NH_2_ from Gln and Asn residues.

The superimposed backbones of both the crystal and NMR structures of AYEdesign and the parent 1AYE crystal structure (Figure 2(a)) demonstrate that RosettaDesign successfully generated a new amino acid sequence that is compatible with the AYEwt fold. This global design accuracy is likely a direct consequence of the highly accurate modeling of side-chain conformations; most side-chains in the core of the AYEdesign NMR and crystal structures are superimposable with those selected in the RosettaDesign computational model (Figure 3). Indeed 73% of all χ_1_ angles, 79% of χ_2_ angles (when χ_1_ is also correct), and 67% of χ_3_ angles (when χ_1_ and χ_2_ are also correct) were recovered accurately (AYEdes_VJQ_chainB compared to AYEdes_model; root-mean-square deviation (Supplementary Data Table S3). When only buried residues are considered, 77%, 100%, and 100% of χ_1_, χ_2_ (when χ_1_ is also correct), and χ_3_ (when χ_1_ and χ_2_ are also correct) angles, respectively, were recovered accurately (Supplementary Data Table S3). A rotamer χ-angle is defined as recovered accurately if the angular difference from the compared χ-angle is less than 40°. These statistics compare favorably to mean rotamer recovery in side-chain repacking experiments of natural proteins using Rosetta (data not shown).

The atomic-level similarity between the RosettaDesign computational model and the experimentally determined high-resolution structures of AYEdesign suggests that specific computationally designed atomic-level interactions were directly responsible for the observed significant increase in thermodynamic stability. We successfully engineered over 10 kcal/mol of increased stability over the wild-type AYE protein, while changing 48 out of 70 residues in the design process. However, in the stabilization of biologically relevant proteins, the aim is often to gain the maximal amount of stability with the minimal number of amino acid substitutions. Could we identify a smaller subset of the AYEdesign mutations that would still yield significant stabilization or were the large number of designed residues synergistically critical for the observed stabilization ? In addition to understanding the specific structural reasons behind the AYEdesign stabilization, this reductionist approach would provide a route to developing and parameterizing an automated computational method for identifying small clusters of stabilizing amino acid mutations.

Using RosettaDesign and structural inspection of the experimentally determined AYEdesign and AYEwt structures, we identified a set of residues likely to contribute to increased stability. We focused on designed residues that improve inter-residue packing (increase in attractive interactions and/or removal of repulsive interactions) and are likely to increase the amount of hydrophobic surface area that is buried upon folding; similar strategies have been employed to stabilize proteins by computational protein design.^15,26^ In order to generalize our conclusions about protein stabilization, we categorized mutations in terms of their potential contribution to inter-helical packing, inter-strand packing, and helix–strand packing. We used RosettaDesign to score different combinations of these mutations in the context of the wild-type protein crystal structure (1AYE). In the design calculations, sets of one to three residues were allowed a binary choice between their AYEwt and AYEdesign sequence identities. All other amino acids were restricted to their wild-type sequence identities, but were allowed to repack.

**Figure 5:**
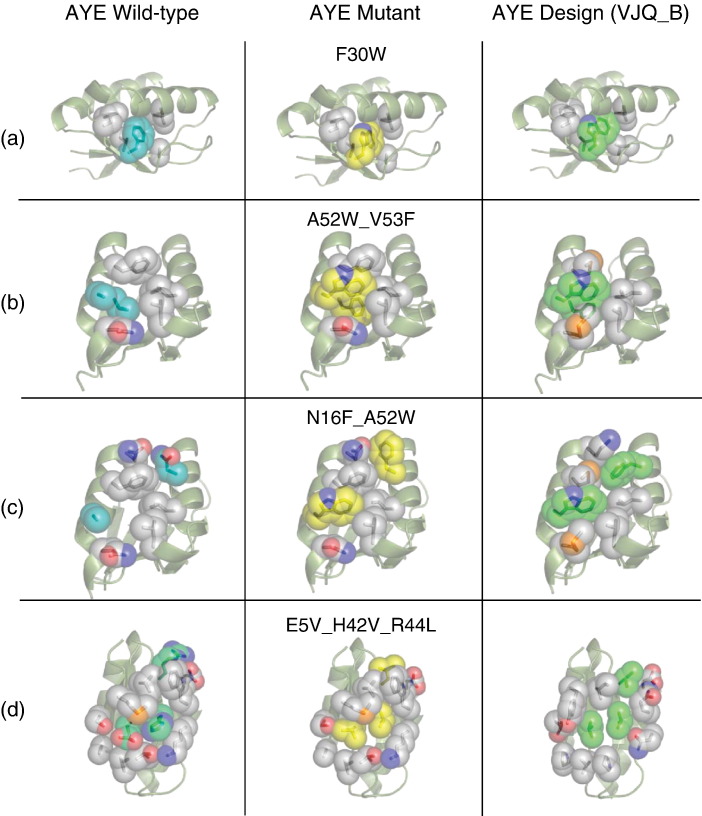
Recapitulation of RosettaDesign stabilization with minimal mutations in AYE. RosettaDesign models of top-scoring AYE mutants (side-chains, yellow) in the context of their AYEwt structural amino acid neighbors (side-chains, CPK) with the AYEwt protein backbone represented in ribbons (olive) are shown in the central column. The corresponding views of the AYEwt (mutated side-chains, cyan) and AYEdes_VJQ chain B(mutated side-chains, green) crystal structures are shown in the left and right columns, respectively. The mutations are labeled above the corresponding illustration in the central column.

For experimental testing, we chose the five lowest-energy two-point and three-point mutants according to the version of RosettaDesign used to select the original AYEdesign sequence (*Rosetta_SmallRadii*). Two additional four-point and five-point mutants that are combinations of structurally-independent mutational clusters from the top-scoring mutants were also selected for experimental characterization to assess additivity in stabilization. Designed mutants N16F_A52W and A52W_V53F are predicted to improve inter-helical packing, E5V_H42V and E5V_H42V_R44L are predicted to improve inter-strand packing, I14V_T40P is predicted to alleviate a helix–strand inter-atomic clash, F30W is predicted to improve helix–strand packing, and E5V_H42V_R44L_F30W and E5V_H42V_R44L_A52W_V53F test combinations of the other mutants. The middle column in Figure 5 shows the RosettaDesign models of the mutants (yellow) in the context of their AYEwt structural amino acid neighbors (colored CPK). The corresponding views of the AYEwt and AYEdes_VJQ crystal structures are shown in the left and right columns, respectively.

**Figure 6:**
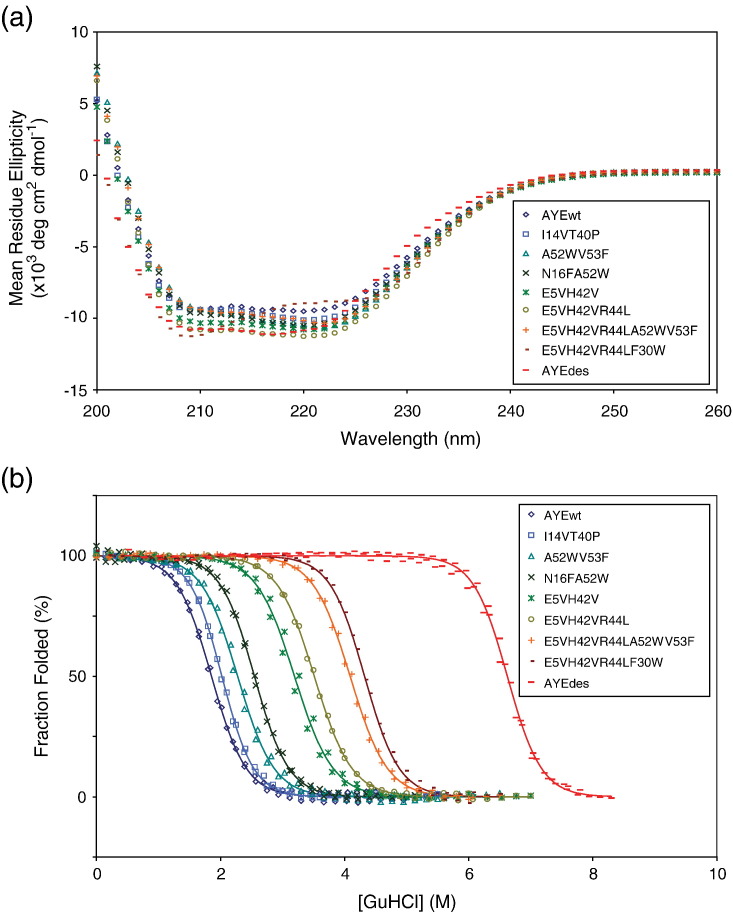
Biophysical characterization of AYE stabilization recapitulation mutants. (a) The far-UV CD spectra of 25 μM AYEwt, seven designed AYE mutants, and AYEdes in 25 mM Tris (pH 8.0), 50 mM NaCl, at 25 °C. (b) The CD signal at 220 nm as a function of GuHCl concentration for all the above proteins at a concentration 5 μM in 25 mMHCl (pH 8.0), 50 mM NaCl at 25 °C.

Site-directed mutagenesis of the AYEwt gene was used to generate the designed mutants described above. Like AYEwt and AYEdes, the mutant proteins were over-expressed in *Escherichia coli*, and purified to ≥95% homogeneity using Ni-affinity chromatography. All mutants were expressed at high levels and were soluble. The far-UV CD scans of all the mutants are identical with AYEwt and AYEdes (Figure 6(a)), suggesting that the mutations did not affect protein secondary structure significantly. Protein stability was assessed by following the guanidine hydrochloride (GuHCl)-induced change of the CD signal. The free energies of unfolding were estimated from the excellent fits of the chemical denaturation data (Figure 6(b)) to a two-state model.

All seven designed mutants were found to be more stable than AYEwt (Table 2). A52W_V53F and I14V_T40P were modestly stabilizing with free energy improvements over wild-type of 0.7 and 0.8 kcal/mol, respectively. The two other two-point mutants, N16F_A52W and E5V_H42V, showed increased stabilization with free energy improvements over wild-type of 1.5 and 2.2 kcal/mol, respectively. The three-point mutant E5V_H42V_R44L and the five-point combination mutant E5V_H42V_R44L_A52W_V53F showed dramatic free-energy improvements of 3.0 kcal/mol and 4.1 kcal/mol, respectively. The most dramatic stabilization was observed with the four-point mutant E5V_H42V_R44L_F30W, which resulted in a free-energy improvement of 5.2 kcal/mol. In contrast, the original AYEdesign achieved 10.3 kcal/mol of stabilization, but 48 residues were changed in the design process.

These results show that the force-field used to successfully design the extremely stable AYEdesign sequence^13^ is successful also in selecting multiple smaller subsets of mutants that still confer significant increases in stability into AYEwt. However, the Rosetta force-field has been through significant changes since the original redesign experiment, and we were interested in evaluating whether attempts at improving conformational sampling and force-field smoothing have resulted in design protocols that retain their successful predictive power.

To redesign the sequence of even a small protein, the size of the sequence–structure space to be searched is enormous.^1–4^ A variety of approximations have been employed to render this problem computationally tractable. The most common is to hold the co-ordinates of protein backbone atoms fixed, and to select residues that stabilize this conformation; this is often referred to as the “inverse folding problem”.^27^ Furthermore, side-chain torsional degrees of freedom are typically restricted to a discrete set of commonly observed values (rotamers).^28^ The limited conformational sampling afforded by these approximations is coarse relative to the spatial variation of the Lennard-Jones potentials used to evaluate the packing of potential protein structure. This sparse sampling of atomistic potentials by design algorithms can lead to severe under-packing of protein cores. It is common practice to address this difficulty by reducing the atomic radii used to evaluate packing,^12^ and in our original redesign experiment^13^ we scaled down the atomic radii in our model to 95% of CHARMM19 values; we call this force-field *Rosetta_SmallRadii* (Table 1).

In parallel with our large-scale natural protein redesign experiment,^13^ we were also applying RosettaDesign to create a novel protein fold, a protein sequence and structure not previously observed in nature. Because it was unlikely that any arbitrarily chosen protein backbone would be designable, it was essential that the design procedure in this case included a search of backbone conformational space in addition to sequence space. Accordingly, we incorporated the backbone optimization component of the high-resolution structure prediction module of Rosetta into *Rosetta_SmallRadii*, such that iterations between sequence and backbone optimization could proceed under the guidance of the same energy function. This protocol was initially used to select five novel-topology or Top sequences for experimental characterization. While all five Top proteins were quite stable and appeared to have the correct α/β secondary structure profiles, they appeared to have somewhat molten cores. Speculating that this was the result of over-packing the protein interior, we increased the atomic radii to values consistent with high-resolution crystal structures. With this modification, we were able to successfully design the Top7 protein; we found it to be folded and extremely stable (Δ*G*° = 13.2 kcal/mol), and the X-ray crystal structure of Top7 showed it to be virtually identical with the design model (C^α^ RMSD = 1.17Å) at atomic-resolution.^19^ We recently showed that *de novo* structure prediction of small protein domains was also improved by the use of this version of the Rosetta force-field,^29^ producing models of heretofore unprecedented accuracy (RMSD <1.5 Å). Consequently, the force-field used for these successes, termed *Rosetta_HardRep* (Table 1), became the default for both protein design and high-resolution structure prediction in Rosetta. It is important to note that both of the applications for which this force-field proved superior incorporate some type of backbone conformational freedom.

To compare the relative predictive ability of the old and new Rosetta force-fields in the context of a fixed protein backbone design simulation, we repeated the AYE mutant design simulations using *Rosetta_SmallRadii* and *Rosetta_HardRep.* The predicted free energies of these mutants relative to the wild-type protein are summarized in Table 2. The *Rosetta_SmallRadii* force-field was able to successfully predict the stabilizing effect of a majority of the designed mutants. In stark contrast, the *Rosetta_HardRep* force-field incorrectly predicted all but two of the mutants to be destabilizing. This supports the idea that tight packing, when described with coarse conformational sampling but evaluated with a standard molecular mechanics potential, can yield spurious atom–atom clashes. We have attempted to reconcile the sampling and the evaluation by altering each separately. In the former case, we have developed a damped variant of the Lennard-Jones potential, and in the latter we have expanded the conformational sampling by introducing limited backbone flexibility.

**Supplementary Data Figure S3:**
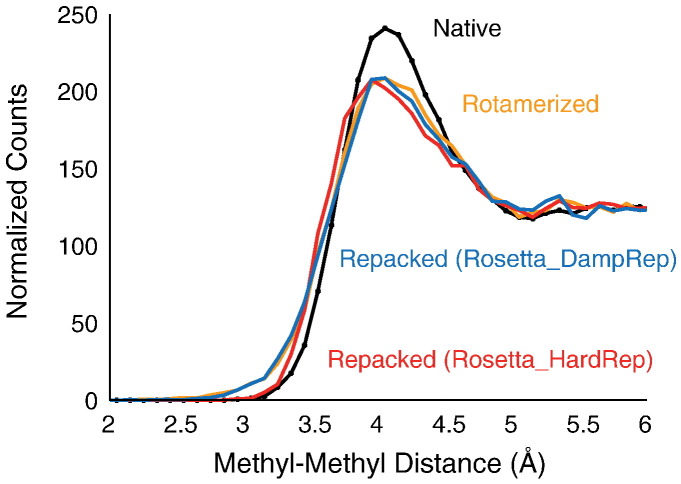


It has been shown that rotamer libraries must be supplemented with a large number of extra dihedral–space conformers to sample adequately a standard Lennard-Jones potential when the backbone is held fixed.^30^ We have taken an alternative approach by selecting a computationally tractable rotamer library (and thus a fixed sampling density in side-chain dihedral space), and adapting our packing potential to that level of sampling. This adaptation is necessary to avoid the spurious clashes that result from a mismatch in resolution between sampling and evaluation. Previously, the primary adaptation considered to reduce clashes is a reduction in atomic radii.^12,13^ This has the danger of also shifting the maxima in atom–atom radial distribution functions (RDFs), resulting in systematic deviations from native structures. We have observed such shifts in large-scale repacking tests (data not shown). To overcome these problems, we developed a new force-field, *Rosetta_DampRep* (Table 1), in which the Lennard-Jones potential was modified such that the atom–atom RDFs of structures repacked under the potential match those resulting from replacing each side-chain in the native structure with the rotamer in a given library that has the lowest heavy-atom coordinate RMSD from the native side-chain. Thus, we attempted to match not the native structure, but the best approximation to the native at a fixed resolution. Atomic radii were varied empirically to ensure that maxima in the RDFs of repacked and approximated structures were in agreement (Supplementary Data Figure S3). Atomic radii were either held fixed (typically for polar atoms) or scaled by a factor of 1.07 (typically for non-polar atoms). It is interesting that, in contrast to common practice, we found that some radii should be increased and none decreased to adapt best to the fixed backbone approximation. Although the scaling term was determined empirically, we speculate that expanded radii may correct for either “overcompression” due to the small but finite attractive component of the Lennard-Jones potential at longer ranges or the omission of thermal effects in the repacking calculations. Finally, we empirically selected a “switch point” on the repulsive side of the Lennard-Jones curve at which the potential changes to a linear functional form, with a slope taken from the tangent at the switch point. A single value (given as a fraction of the distance to the potential minimum) was determined for all atom types, and was selected to match the RDFs between repacked and approximated native structures at distances less than the maximum. Although the *Rosetta_DampRep* potential was constructed to match RDFs, we have observed that it also yields improved performance in side-chain repacking applications (Supplementary Data Table S4). We repeated the AYE mutants design simulations using *Rosetta_DampRep*, and observed that, similar to *Rosetta_SmallRadii*, this new force-field successfully predicted the stabilizing effect of a majority of the mutants (Table 2). We were thus able to observe good design predictions in a fixed-backbone context using two different methods for damping the computational evaluation of atomic-overlap in Rosetta; either scaling down atomic radii or explicitly damping the repulsive component of the Lennard-Jones potential.

As an alternate approach to using potentials with damped repulsive terms, we tested whether the *Rosetta_HardRep* potential could be used to successfully predict the stability of the AYE mutants if the protein backbone and side-chains were allowed to relax following mutation. The flexible backbone protocol, *Rosetta_FlexBB* (Table 1), begins with gradient-based minimization of the backbone and side-chain torsion angles in the wild-type structure. Mutations are modeled onto the relaxed wild-type structure and repacked along with neighboring residues to identify low-energy rotamers. Following repacking, the backbone and side-chain torsion angles are minimized once more. The energies of the relaxed wild-type and mutated structures are compared to calculate the change in protein stability. In general, the protein structure does not vary dramatically with this protocol, backbone deviations are typically less than 0.4 Å RMSD. Independent simulations do not produce identical structures and energies; therefore, 100 simulations were performed for each mutation and the lowest energy result was used for comparison. We observed that, similar to the fixed-backbone *Rosetta_SmallRadii* and *Rosetta_DampRep* force-fields, *Rosetta_FlexBB* was able to successfully predict the stabilizing effect of a majority of the AYE mutations (Table 2).

Despite the overall success of these predictions, the design search space was restricted in this test, since the program was given only a binary choice between the original AYEwt and AYEdesign sequences. For a true evaluation of design prediction, all amino acids should be allowed at the design positions. Accordingly, the protocol with the best ΔΔ*G*° prediction for AYEdes, *Rosetta_DampRep*, was used to redesign the mutated residues in the seven mutants described above, allowing all 20 amino acids to be chosen at those positions. Table 3 shows that RosettaDesign predominantly designs either the same or similar amino acids as those picked in the binary choice experiment; E5V, I14T, F30W, T40P, H42V, and A52W are identical, and N16W and V53Y are similar types of mutations (N16F and V52F in AYEdesign). Only R44K does not match the AYEdesign trend.

It is instructive to compare the results of our work with other studies of this protein, since others have used AYEwt as a subject for rational stabilization attempts. Villegas *et al*.^31^ mutated surface-exposed residues on the two helices to improve predicted helical propensity, and reported that two four-point mutants (one set per helix) stabilized the protein by 1.1 kcal/mol and 1.5 kcal/mol. A combined eight-point mutant stabilized the protein by 2.8 kcal/mol. Table 2 shows that RosettaDesign was also able to successfully predict the stabilizing effects of two out of three of these mutants.

## Conclusion

By solving the crystal and NMR structures of AYEdesign, we have demonstrated the high-resolution accuracy of our computational protein design methodology, RosettaDesign. A comparison of the experimentally determined structures and our computational model showed that the extreme thermodynamic stabilization of AYEdes was a direct consequence of atomically accurate modeling of both backbone and side-chain conformations. We used the information gleaned from these structural studies to identify small clusters of residues that can independently provide significant thermodynamic stabilization, and showed that RosettaDesign can successfully predict the stabilizing effect of these mutations. Finally, we compared different force-fields and approaches to computing the free-energy change associated with these stabilizing mutations, and found that good recapitulation with fixed backbone models and coarse sampling around side-chain rotamers requires either reduced radii or damped repulsion terms, while the current, more accurate Rosetta force-field yields good predictions when used with explicit modeling of backbone flexibility.

## Materials and Methods

### Protein expression and purification

The computationally designed amino acid sequence of AYEdesign has been reported.^13^ Two different expression constructs that contain the AYEdesign sequence were prepared.

The first construct (used for crystallography) was a fusion construct in a pET3a-based vector consisting of an N-terminal His_6_ tag, the 70 residues of the AYEdesign sequence, a 15-residue linker containing a TEV protease cleavage site, and the C-terminal 62 residues of structural genomics target sequence Lmaj000047 (geneDB identifier LmjF25.2320). This construct was prepared to leverage the excellent stability and solubility of AYEdesign to potentially improve the solubility of unrelated proteins that are tagged to it. Cleavage of the expressed fusion protein by TEV protease should yield one chain (AYEdes_VJQ) with the N-terminal His_6_ tag, the AYEdesign sequence, and nine C-terminal linker residues from the TEV cleavage site, and another chain with six N-terminal linker residues from the TEV cleavage site and the structural genomics target protein. The fusion protein was expressed in *E. coli* BL21 (DE3) in the presence of selenomethionine as described,^32^ and purified using a Ni-NTA column (Qiagen). The column was washed with His-tagged TEV protease with the intent of releasing only the target Lmaj000047 fragment. However, for unknown reasons, both cleavage fragments were released from the column and inadvertently carried forward into crystallization trials. In any case, the AYEdes_VJQ fragment crystallized preferentially and its structure was determined *de novo* using multiple-wavelength anomalous diffraction (MAD).

The second construct (used for NMR spectroscopy and thermodynamic measurements) was in a pet29b-based vector consisting of an N-terminal His_6_ tag followed by the 70 residues of the AYEdesign sequence. This construct (AYEdes), was expressed in *E. coli* BL21 (DE3) in LB or M9 minimal medium supplemented with appropriately isotope-labeled NH_4_Cl and glucose, as needed, and purified by Ni-affinity chromatography followed by gel-filtration chromatography. Purified samples contained no impurity that was detectable by SDS-PAGE.

The wild-type AYE construct (AYEwt) in a pet29b-based vector was used essentially as described,^21^ except the C-terminal His_6_ tag was moved to the N terminus by PCR subcloning, to match the AYEdes construct. All mutants were generated in the context of this construct using the Quick Change Site-Directed mutagenesis kit (Stratagene).

### Crystallographic structure determination

Crystals of AYEdes_VJQ grew from sitting drops containing 1 μl of protein solution (6.9 mg/ml protein in 20 mM Hepes (pH 7.5), 0.5 M NaCl, 2 mM β-mercaptoethanol, 5% (v/v) glycerol), 1 μl of crystallization buffer (20% (w/v) PEG 1000, 40 mM CaCl_2_, 100 mM sodium acetate), and 1 μl of crystallization buffer containing microcrystalline seeds from earlier crystallization trials.

Diffraction data at three wavelengths were collected from a single crystal on beamline 8.2.1 at the Advanced Light Source (ALS). Data were integrated and scaled using the HKL2000 package.^33^ Essentially the entire backbone of the two monomers in the crystal asymmetric unit was auto-traced by the program RESOLVE,^34^ on the basis of initial phases derived from four Se sites identified automatically by SHELXD.^35^ Automated assignment and fitting of protein side-chains failed utterly, since at that point the sequence was still mistakenly expected to match that of Lmaj000047. Manual inspection of the experimentally phased electron density maps easily assigned identities for residues making up the eight-residue sequence MVEWFLEM spanning the two SeMet sites in each chain. This characteristic sequence fragment revealed the true identity of the structure to be the AYEdes design sequence plus the nine linker residues proximal to the TEV cleavage site (AYEdes_VJQ). The remaining side-chains were placed using the real-space fit and refine mode of Xfit.^36^ Only the first three residues from the linker sequence in each chain were well-ordered; hence, the final crystallographic model contains 73 residues per chain. The structure was refined at 2.1 Å resolution using the program REFMAC5,^37^ yielding standard residuals *R*_work_ and *R*_free_ of 0.20 and 0.27, respectively (Supplementary Data Table S1). The stereochemistry and fit to density of the final model were validated using MolProbity^38^ and Coot.^39^ Of 142 (ϕ,ψ) dihedral angle pairs, 139 are in favored regions of backbone conformational space, while the remaining three residues are in allowed regions.

### NMR structure determination

Freshly purified samples of AYEdes were concentrated to ∼1 mM by centrifugation and were prepared for NMR studies in 8% (v/v) ^2^H_2_O or ∼100% ^2^H _2_O containing 50 mM potassium phosphate (pH 7.0) and 100 mM KCl. NMR experiments were recorded at 298 K on a Bruker DRX 500 and DMX 750 MHz as well as on a Bruker 500 MHz equipped with cryo-probe (at NMRFAM) and Varian 600 MHz spectrometers (at PNNL). Combinations of standard triple-resonance experiments (HNCO, HNCA, HN(CO)CACB, HNCACB, and HCCH-TOCSY)^40^ were used to obtain nearly complete assignments for 72 residues. Heteronuclear ^13^C and ^15^N-edited 3D and homonuclear 2D NOESY experiments collected with mixing times of 100 ms were used to obtain structural restraints. Intra-molecular restraints were obtained from ^13^C-filtered chirp-NOESY data collected at PNNL with a mixing time of 100 ms on the 600 MHz Varian spectrometer equipped with a cryo-probe. Spectra were processed using NMRPipe,^41^ and analyzed with Sparky†. ^1^H,^15^N-Heteronuclear NOE, *T*_1_ and *T*_2_ relaxation experiments were all collected on the Bruker DRX 500 MHz spectrometer and analyzed using ModelFree.^42^ NOE assignments and structure calculations for the AYEdes monomeric subunit were performed initially using combined automated and manual methods in CYANA.^24^ Assigned inter-molecular NOE restraints were duplicated for chains A and B of the AYEdes symmetric homodimer and intra-molecular NOE restraints were derived from ^13^C-filtered chirp-NOESY data. Torsion angle restraints were included for ϕ/ψ angles according to TALOS predictions,^43^ and hydrogen bonding constraints were derived from amide ^2^H_2_O protection data. Structure calculations for AYEdes were completed with CYANA v2.1 and visualized using MOLMOL.^44^ Analysis with PROCHECK found 100% of the residues for AYEdes in allowed regions of the Ramachandran plot.^45^ Structural statistics from 20/100 lowest energy structures (chains A and B) are provided (Supplementary Data Table S2).

### Size-exclusion (gel-filtration) chromatography

Size-exclusion chromatography was carried out using an analytical Superdex-75 column (Amersham Pharmacia) with the Pharmacia FPLC system (GP-250 gradient programmer, P-500 Pump). Protein samples at concentrations used for NMR (600 μM–1.2 mM) or CD (5–100 μM) were equilibrated in 25 mM Tris– HCl (pH 8.0), 20 mM EDTA, 50 mM NaCl at 25 °C, and run on the Superdex-750 column at a flow-rate of 1 ml/min.

### Analytical ultra-centrifugation

Sedimentation equilibrium studies on AYEdes were conducted in a Beckman XL-A analytical ultracentrifuge using six-channel 12 mm Epon charcoal-filled centerpieces. All scans were conducted at 20 °C using an absorbance wavelength of 280 nm at rotor speeds of 25,000 rpm, 35,000 rpm, and 45,000 rpm. AYEdes concentrations were determined from a scan at 3000 rpm to be 13 μM, 33 μM, and 50 μM. Data were collected in 25 mM Tris–HCl (pH 8.0), 50 mM NaCl with and without 20 mM EDTA. The effect of EDTA on the associative state of AYEdes was negligible. Equilibration for 8 h was deemed sufficient by identical absorbance scans collected after 6 h and 8 h at each speed.

The UltraScan software package was used for data analysis as well as deriving solvent density and partial specific volume parameters‡. The weight-averaged molecular mass, *M*_W_, was determined for individual equilibrium scans by fitting to a single ideal species model using non-linear least-squares analysis. Residuals to the fit were random, and the fitted values for the baseline offset agreed well with values determined using the meniscus-depletion method. Next, global fits were performed for each protein concentration across the three speeds to determine *M*_W_ at each concentration. A monomer–dimer equilibrium model was used to determine the dissociation constant. Confidence limits were determined by Monte Carlo analysis with UltraScan.

### Circular dichroism (CD)

CD data were collected on an Aviv 62A DS spectrometer. Far-UV CD wavelength scans (260–195 nm) at 25 °C were collected in a 1 mm path-length cuvette. Guanidinium hydrochloride (GuHCl)-induced protein denaturation was followed by the change in ellipticity at 220 nm in a 1 cm path-length cuvette, using a Microlab titrator (Hamilton) for denaturant mixing. Temperature was maintained at 25 °C with a Peltier device. All CD data were converted to mean residue ellipticity. To obtain a value for Δ*G*_U_^H2O^, the denaturation curves were fit by non-linear least-squares analysis using a linear extrapolation model.^46^

### Computational procedure

Our method for computational protein design, RosettaDesign, has been described in detail.^13,19^ In brief, RosettaDesign contains two main components; an energy function that ranks the relative fitness of amino sequences for a given protein structure and a Monte Carlo optimization procedure for rapidly searching sequence space. The energy function is a linear combination of a 6-12 Lennard-Jones potential, the Lazaridis–Karplus implicit solvation model,^47^ an empirical hydrogen bonding potential,^48^ backbone-dependent rotamer probabilities,^49^ amino acid probabilities for particular regions of ϕ/ψ space, and a simple electrostatics pseudo-energy derived from the distance distributions of polar residues in the PDB.^50^ In addition, each amino acid has a unique reference energy that provides an implicit treatment of the unfolded state and enforces a native-like sequence composition. Weights for the various energy terms were determined as described.^51^ Briefly, all rotamers from a backbone-dependent library are placed at each position in a set of proteins. Each energy component is calculated for each rotamer with the remainder of the protein held fixed. The energy terms form the coefficients of a matrix; an optimal vector of weights is obtained such that the energy gap between native and non-native rotamers is maximized. These weights are then used to redesign fully the training set of proteins. The weights are again optimized, now using the redesigned proteins, rather than the native proteins, as the context in which the energy matrix is determined. The procedure is iterated through ∼5 cycles of weight optimization and full redesign before the weights converge. This process compensates for the fact that the weight determination step makes a linearizing approximation to the full design problem when it calculates rotamer energies in an otherwise unchanged background.

Four variants of the general RosettaDesign force-field were employed in this study (Table 1). In the version of RosettaDesign used to select the original AYEdesign sequence (*Rosetta_SmallRadii*),^13^ the atomic radii were scaled by 0.95 relative to standard CHARMM 19 radii. The damped repulsive variant of RosettaDesign (*Rosetta_DampRep*) differs from standard RosettaDesign in its treatment of the Lennard-Jones potential in the repulsive region (where atom–atom energies are greater than zero). At distances less than a specified fraction of the energy minimum, the potential takes on a linear form, with its slope selected to match that of the Lennard-Jones potential at that distance. Additionally, atomic radii are scaled by a constant factor; a factor of 1.07 was found empirically to improve agreement between the atom–atom distance distribution maxima observed in native crystal structures and the same structures when side-chain positions were repacked using RosettaDesign. Both *Rosetta_SmallRadii* and *Rosetta_DampRep* keep the co-ordinates of the protein backbone fixed during the design simulation. In a third variant of RosettaDesign, the protein backbone was allowed to relax following a mutation. This protocol (*Rosetta_FlexBB*) begins with relaxing the wild-type structure with gradient-based minimization of side-chain and backbone torsion angles using an energy function that has full-size radii and a standard representation of the Lennard-Jones potential. This relaxed structure is used to calculate the energy of the wild-type sequence and is used as the template for making mutations. The energy of the mutant structure is determined by repacking the residues surrounding the site of mutation followed by gradient-based minimization of backbone and side-chain torsion angles. As a control for the above RosettaDesign variants, a fourth protocol (*Rosetta_HardRep*) uses the *Rosetta_FlexBB* force-field with standard atomic radii and Lennard-Jones potential, while keeping the protein backbone fixed during design simulations.

### PDB accession codes

X-ray coordinates and structure factors have been deposited with the PDB as accession code 1vjq. NMR Coordinates and experimental constraint files have been deposited with the PDB as accession code 2gjf.

## Figures and Tables

**Table 1:** Summary of RosettaDesign force-field variants

Force-field variant	Relative radii size	Repulsive treatment	Backbone flexibility
*HardRep*	–	*r*^12^	No
*SmallRadii*	< *HardRep*	*r*^12^	No
*DampRep*	> *HardRep*	Damped *r*^12^	No
*FlexBB*	= *HardRep*	*r*^12^	Yes

See Materials and Methods for details.

**Table 2:**
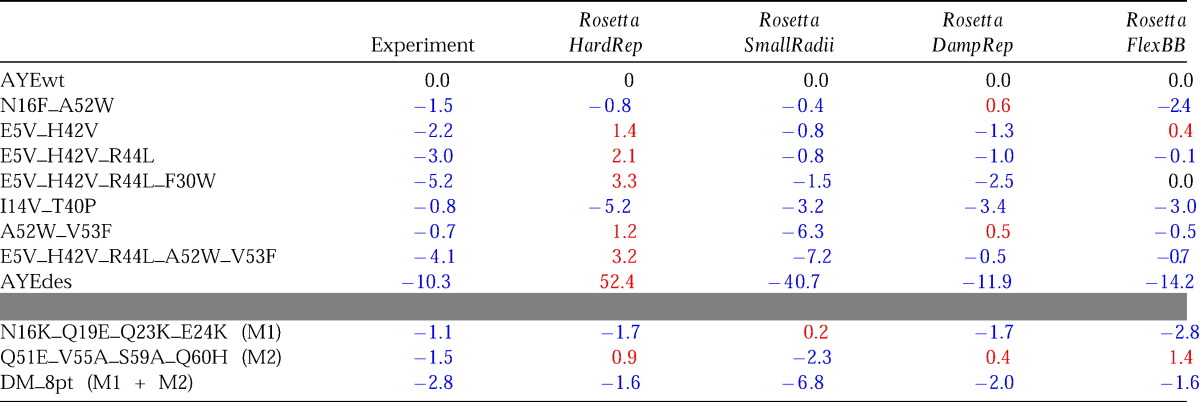
Observed and computed ΔΔ*G* values (kcal/mol) for AYE mutants

**Table 3:**
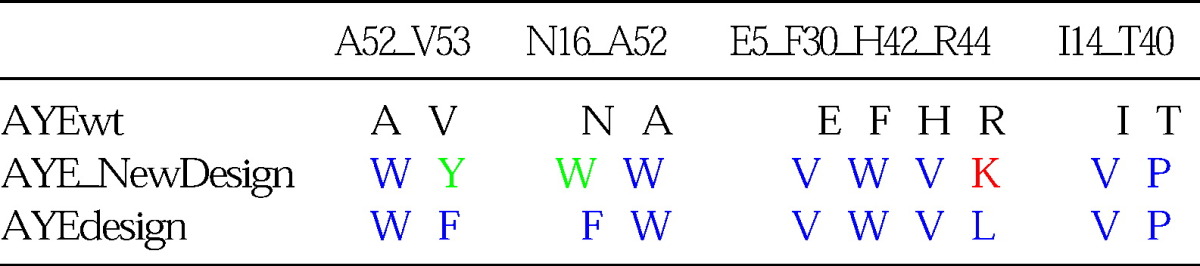
*Rosetta_DampRep* design predictions for AYE stabilization recapitulation clusters

## Footnotes

† http://www.cgl.ucsf.edu/home/sparky/

‡ www.ultrscan.uthscsa.edu
