# Supplementary material for: High-resolution Structural and Thermodynamic Analysis of Extreme Stabilization of Human Procarboxypeptidase by Computational Protein Design
Source: J Mol Biol. 2007 Mar 2;366(4):1209–21. doi: 10.1016/j.jmb.2006.11.080 (PMC3764424; doi:10.1016/j.jmb.2006.11.080)
Supplement: Supplementary Data Table [file mmc1.doc]

**Table S1:** AYEdes_VJQ crystallographic data collection and refinement Statistics

| Space Group |  |
| --- | --- |
| Unit Cell (Å) | a=61.44 b=65.04 c=39.20 |
| Wavelength (Å) | 0.97954 |
| Resolution Range (Å) | 44.7 - 2.10 (2.18 - 2.10) |
| Unique reflections | 8236 (314) |
| Redundancy | 11.1 (2.7) |
| Completeness | 85.0 (32.9) |
| *I*/σ(*I*) | 11.7 (2.2) |
|  | 0.108 (0.375) |
|  | 0.196 |
|  | 0.269 |
| RMSD bond lengths (Å) | 0.012 |
| RMSD bond angles (°) | 1.241 |
| Number of protein atoms | 1174 |
| Number of non-protein atoms | 65 |
|  |  |
|  |  |

**Table S2.** NMR and refinement statistics for AYEdes

| **NMR distance & dihedral constraints** |  |
| --- | --- |
| Distance constraints* |  |
| Total NOE | 1824 |
| Intra-residue | 391 |
| Inter-residue | 1333 |
| Sequential (|i-j| = 1) | 442 |
| Medium-range (|i-j| < 4) | 401 |
| Long-range (|i-j > 5) | 567 |
| Long-range (dimer interface) | 23 |
| Hydrogen bonds | 25 |
| Total dihedral angle restraints | 104 |
| Phi | 52 |
| Psi | 52 |
|  |  |
| **Structure Statistics** |  |
| Violations (mean and s.d.) |  |
| Distance constraints (Å) | 0.0060 ± 0.0003 |
| Dihedral angle constraints (º) | 0.2025 ± 0.0320 |
| Max. dihedral angle violation (º) | 1.47 |
| Max. distance constraint violation (Å) | 0.19 |
| Average pairwise r.m.s.d.** (Å) |  |
| Backbone |  |
| Dimer | 0.57 ± 0.18 |
| Monomer A | 0.26 ± 0.07 |
| Monomer B | 0.28 ± 0.05 |
| Heavy Atom |  |
| Dimer | 1.09 ± 0.11 |
| Monomer A | 0.92 ± 0.07 |
| Monomer B | 0.99 ± 0.08 |
| Ramachandran Plot Statistics*** (%) |  |
| Most Favored Regions | 87.1 |
| Additionally Allowed Regions | 12.8 |
| Generously Allowed Regions | 0.0 |
| Disallowed Regions | 0.0 |

Final 20/100 structures of the AYEdes (Gly1-Glu72) homo-dimer were selected

based on lowest Cyana ^1^ target functions

*Distance constraints are reported for each monomeric sub-unit of AYEdes

**Pairwise r.m.s.d. calculated over ordered residues (Lys3-Glu71)

***Ramachandran plot statistics obtained with ProCheck ^2^

**Table S3.** Rotamer recovery statistics for AYEdesigns

Rotamer Recovery for AYEdes_model.pdb vs. AYEdes_model.pdb

------ALL------ ----BURIED----- ----MIDDLE----- ----SURFACE----

#Cor #Tot Frac #Cor #Tot Frac #Cor #Tot Frac #Cor #Tot Frac

CHI_1 64 64 1.00 14 14 1.00 29 29 1.00 21 21 1.00

CHI_12 44 44 1.00 10 10 1.00 18 18 1.00 16 16 1.00

CHI_123 19 19 1.00 1 1 1.00 7 7 1.00 11 11 1.00

Rotamer Recovery for AYEdes_vjqA.pdb vs. AYEdes_model.pdb

------ALL------ ----BURIED----- ----MIDDLE----- ----SURFACE----

#Cor #Tot Frac #Cor #Tot Frac #Cor #Tot Frac #Cor #Tot Frac

CHI_1 41 64 0.64 11 13 0.85 18 24 0.75 12 27 0.44

CHI_12 21 29 0.72 9 9 1.00 6 9 0.67 6 11 0.55

CHI_123 4 7 0.57 1 1 1.00 2 2 1.00 1 4 0.25

Rotamer Recovery for AYEdes_vjqB.pdb vs. AYEdes_model.pdb

------ALL------ ----BURIED----- ----MIDDLE----- ----SURFACE----

#Cor #Tot Frac #Cor #Tot Frac #Cor #Tot Frac #Cor #Tot Frac

CHI_1 47 64 0.73 10 13 0.77 24 29 0.83 13 22 0.59

CHI_12 27 34 0.79 8 8 1.00 14 15 0.93 5 11 0.45

CHI_123 6 9 0.67 1 1 1.00 4 6 0.67 1 2 0.50

Rotamer Recovery for AYEdes_nmrA.pdb vs. AYEdes_model.pdb

------ALL------ ----BURIED----- ----MIDDLE----- ----SURFACE----

#Cor #Tot Frac #Cor #Tot Frac #Cor #Tot Frac #Cor #Tot Frac

CHI_1 42 64 0.66 10 13 0.77 13 20 0.65 19 31 0.61

CHI_12 23 30 0.77 7 7 1.00 8 8 1.00 8 15 0.53

CHI_123 4 7 0.57 0 1 0.00 1 1 1.00 3 5 0.60

Rotamer Recovery for AYEdes_nmrB.pdb vs. AYEdes_model.pdb

------ALL------ ----BURIED----- ----MIDDLE----- ----SURFACE----

#Cor #Tot Frac #Cor #Tot Frac #Cor #Tot Frac #Cor #Tot Frac

CHI_1 38 64 0.59 11 14 0.79 13 23 0.57 14 27 0.52

CHI_12 22 27 0.81 6 7 0.86 8 8 1.00 8 12 0.67

CHI_123 3 9 0.33 0 1 0.00 2 2 1.00 1 6 0.17

LEGEND

BURIED : neighbours > 19

MIDDLE : neighbours < 20 AND > 13

SURFACE : neighbours < 14

#Cor : Number of rotamers with correct chi

#Tot : Number of rotamers with possible correct chi

Frac : Fraction of correct chi

CHI_1 : Stats for all rotamers with chi1

CHI_12 : Stats for all rotamers with chi2, IF correct chi1

CHI_123 : Stats for all rotamers with chi3, IF correct chi1 AND chi2

DEFINITIONS:

Neighbours are all residues with c-beta within 14 angstroms of the reference residue c-beta. A chi-angle is defined as correct if the angular difference from the compared chi-angle is less than 40 degrees. The 13 buried residues are PHE4, ILE6, PRO8, GLN13, VAL14, LEU17, LEU20, PRO34, VAL41, ILE43, ILE45, PHE53, and LEU57.

**Table S4.** Sidechain χ-angle recovery improvement with *Rosetta_DampRep* potential

| Amino Acid | Sample size | χ_1_ correct  (%)/[Δ]^†^ | χ_1+2_ correct  (%)/[Δ]^†^ | χ_1+2+3_ correct  (%)/[Δ]^†^ | χ_1+2+3+4_ correct (%)/[Δ]^†^ |
| --- | --- | --- | --- | --- | --- |
| ASN | 3171 | 88 [+1] | 75 [+3] |  |  |
| ASP | 4471 | 86 [+0] | 76 [+1] |  |  |
| ARG | 3409 | 83 [+1] | 70 [+1] | 50 [+2] | 38 [+2] |
| CYS | 863 | 93 [+1] |  |  |  |
| GLN | 2770 | 82 [+0] | 65 [+1] | 47 [+2] |  |
| GLU | 4825 | 76 [+0] | 61 [+1] | 37 [+1] |  |
| HIS | 1805 | 91 [+1] | 64 [+2] |  |  |
| ILE | 4144 | 96 [+1] | 88 [+3] |  |  |
| LEU | 6506 | 95 [+1] | 87 [+3] |  |  |
| LYS | 4568 | 79 [+0] | 66 [+1] | 51 [+1] | 35 [+1] |
| MET | 1634 | 88 [+0] | 78 [+0] | 63 [+0] |  |
| PHE | 3006 | 96 [+1] | 93 [+2] |  |  |
| PRO | 3335 | 85 [+2] |  |  |  |
| SER | 4138 | 74 [+1] |  |  |  |
| THR | 4024 | 92 [+0] |  |  |  |
| TRP | 998 | 94 [+2] | 85 [+3] |  |  |
| TYR | 2516 | 96 [+2] | 91 [+2] |  |  |
| VAL | 5481 | 93 [+1] |  |  |  |
| ALL | 61664 | 87 [+0] | 77 [+2] | 48 [+2] | 37 [+2] |

^†^ Values in brackets are the change with respect to the standard Lennard‑Jones potential implemented in Rosetta (*Rosetta_HardRep*). The weights for both potentials were optimized for recovery of sidechain conformation (as opposed to sequence recovery) on a training set separate from the test set.

**References**

1. Guntert, P. (2003). Automated NMR protein structure calculation. *Progress In Nuclear Magnetic Resonance Spectroscopy* **43**, 105-125.

2. Laskowski, R. J., Macarthur, M. W., Moss, D. S. & Thornton, J. M. (1993). PROCHECK: a program to check the stereochemical quality of protein structures. *J. Appl. Crystall.* **26**, 283 - 291.
