# Supplementary material for: High-resolution Structural and Thermodynamic Analysis of Extreme Stabilization of Human Procarboxypeptidase by Computational Protein Design
Source: J Mol Biol. 2007 Mar 2;366(4):1209–21. doi: 10.1016/j.jmb.2006.11.080 (PMC3764424; doi:10.1016/j.jmb.2006.11.080)
Supplement: Supplementary Data Table [file mmc2.pdf]

```
REMARK  AYEdes_model: redesign of 1AYE.PDB
REMARK  RosettaDesign model for redesigned procarboxypeptidase A
ATOM      1  N   LYS     1      12.548 -15.304   3.221  1.00  0.00
ATOM      2  CA  LYS     1      13.304 -14.095   3.578  1.00  0.00
ATOM      3  C   LYS     1      14.451 -14.452   4.511  1.00  0.00
ATOM      4  O   LYS     1      14.290 -15.250   5.446  1.00  0.00
ATOM      5  CB  LYS     1      12.383 -13.059   4.225  1.00  0.00
ATOM      6  CG  LYS     1      11.280 -12.541   3.312  1.00  0.00
ATOM      7  CD  LYS     1      10.418 -11.505   4.019  1.00  0.00
ATOM      8  CE  LYS     1       9.318 -10.983   3.106  1.00  0.00
ATOM      9  NZ  LYS     1       8.452  -9.986   3.791  1.00  0.00
ATOM     10  H   LYS     1      12.034 -16.126   2.978  1.00  0.00
ATOM     11 1HZ  LYS     1       7.737  -9.666   3.153  1.00  0.00
ATOM     12 2HZ  LYS     1       8.018 -10.414   4.597  1.00  0.00
ATOM     13 3HZ  LYS     1       9.014  -9.201   4.089  1.00  0.00
ATOM     14  HA  LYS     1      13.745 -13.657   2.682  1.00  0.00
ATOM     15 1HB  LYS     1      11.937 -13.531   5.101  1.00  0.00
ATOM     16 2HB  LYS     1      13.011 -12.226   4.543  1.00  0.00
ATOM     17 1HG  LYS     1      11.742 -12.092   2.432  1.00  0.00
ATOM     18 2HG  LYS     1      10.660 -13.383   3.006  1.00  0.00
ATOM     19 1HD  LYS     1       9.971 -11.968   4.900  1.00  0.00
ATOM     20 2HD  LYS     1      11.055 -10.677   4.329  1.00  0.00
ATOM     21 1HE  LYS     1       9.785 -10.521   2.237  1.00  0.00
ATOM     22 2HE  LYS     1       8.711 -11.829   2.784  1.00  0.00
ATOM     23  N   THR     2      15.605 -13.849   4.261  1.00  0.00
ATOM     24  CA  THR     2      16.778 -14.110   5.082  1.00  0.00
ATOM     25  C   THR     2      17.286 -12.869   5.764  1.00  0.00
ATOM     26  O   THR     2      17.207 -11.778   5.208  1.00  0.00
ATOM     27  CB  THR     2      17.926 -14.714   4.252  1.00  0.00
ATOM     28  OG1 THR     2      17.498 -15.953   3.671  1.00  0.00
ATOM     29  CG2 THR     2      19.144 -14.963   5.128  1.00  0.00
ATOM     30  H   THR     2      15.671 -13.200   3.490  1.00  0.00
ATOM     31  HG1 THR     2      17.252 -16.565   4.369  1.00  0.00
ATOM     32  HA  THR     2      16.521 -14.806   5.881  1.00  0.00
ATOM     33  HB  THR     2      18.189 -14.020   3.454  1.00  0.00
ATOM     34 1HG2 THR     2      19.944 -15.389   4.524  1.00  0.00
ATOM     35 2HG2 THR     2      19.478 -14.021   5.562  1.00  0.00
ATOM     36 3HG2 THR     2      18.882 -15.657   5.925  1.00  0.00
ATOM     37  N   ILE     3      17.794 -13.032   6.980  1.00  0.00
ATOM     38  CA  ILE     3      18.331 -11.901   7.715  1.00  0.00
ATOM     39  C   ILE     3      19.841 -12.139   7.683  1.00  0.00
ATOM     40  O   ILE     3      20.331 -13.188   8.125  1.00  0.00
ATOM     41  CB  ILE     3      17.758 -11.831   9.142  1.00  0.00
ATOM     42  CG1 ILE     3      16.265 -11.492   9.102  1.00  0.00
ATOM     43  CG2 ILE     3      18.517 -10.807   9.971  1.00  0.00
ATOM     44  CD1 ILE     3      15.571 -11.632  10.437  1.00  0.00
ATOM     45  H   ILE     3      17.807 -13.952   7.398  1.00  0.00
ATOM     46  HA  ILE     3      18.119 -10.969   7.193  1.00  0.00
ATOM     47  HB  ILE     3      17.845 -12.812   9.608  1.00  0.00
ATOM     48 1HG2 ILE     3      18.098 -10.770  10.976  1.00  0.00
ATOM     49 2HG2 ILE     3      19.567 -11.090  10.026  1.00  0.00
ATOM     50 3HG2 ILE     3      18.430  -9.825   9.506  1.00  0.00
ATOM     51 1HG1 ILE     3      16.175 -10.464   8.752  1.00  0.00
ATOM     52 2HG1 ILE     3      15.800 -12.163   8.379  1.00  0.00
ATOM     53 1HD1 ILE     3      14.517 -11.376  10.329  1.00  0.00
ATOM     54 2HD1 ILE     3      15.659 -12.661  10.788  1.00  0.00
ATOM     55 3HD1 ILE     3      16.033 -10.962  11.160  1.00  0.00
```

```
ATOM     56  N   PHE     4      20.562 -11.190   7.090  1.00  0.00
ATOM     57  CA  PHE     4      22.013 -11.275   6.971  1.00  0.00
ATOM     58  C   PHE     4      22.732 -10.345   7.921  1.00  0.00
ATOM     59  O   PHE     4      22.249  -9.256   8.226  1.00  0.00
ATOM     60  CB  PHE     4      22.450 -10.970   5.537  1.00  0.00
ATOM     61  CG  PHE     4      21.787 -11.836   4.504  1.00  0.00
ATOM     62  CD1 PHE     4      20.556 -11.484   3.970  1.00  0.00
ATOM     63  CD2 PHE     4      22.391 -13.004   4.065  1.00  0.00
ATOM     64  CE1 PHE     4      19.945 -12.279   3.020  1.00  0.00
ATOM     65  CE2 PHE     4      21.783 -13.800   3.114  1.00  0.00
ATOM     66  CZ  PHE     4      20.558 -13.437   2.591  1.00  0.00
ATOM     67  H   PHE     4      20.088 -10.383   6.709  1.00  0.00
ATOM     68  HD1 PHE     4      20.072 -10.567   4.308  1.00  0.00
ATOM     69  HE1 PHE     4      22.269 -14.715   2.776  1.00  0.00
ATOM     70  HZ  PHE     4      20.077 -14.066   1.843  1.00  0.00
ATOM     71  HE2 PHE     4      22.269 -14.715   2.776  1.00  0.00
ATOM     72  HD2 PHE     4      23.359 -13.290   4.478  1.00  0.00
ATOM     73  HA  PHE     4      22.347 -12.280   7.231  1.00  0.00
ATOM     74 1HB  PHE     4      22.207  -9.939   5.282  1.00  0.00
ATOM     75 2HB  PHE     4      23.523 -11.126   5.432  1.00  0.00
ATOM     76  N   VAL     5      23.862 -10.804   8.433  1.00  0.00
ATOM     77  CA  VAL     5      24.652  -9.993   9.336  1.00  0.00
ATOM     78  C   VAL     5      25.805  -9.563   8.447  1.00  0.00
ATOM     79  O   VAL     5      26.504 -10.397   7.864  1.00  0.00
ATOM     80  CB  VAL     5      25.085 -10.791  10.580  1.00  0.00
ATOM     81  CG1 VAL     5      26.143 -10.026  11.362  1.00  0.00
ATOM     82  CG2 VAL     5      23.885 -11.092  11.465  1.00  0.00
ATOM     83  H   VAL     5      24.178 -11.732   8.191  1.00  0.00
ATOM     84  HA  VAL     5      24.100  -9.111   9.663  1.00  0.00
ATOM     85  HB  VAL     5      25.492 -11.751  10.261  1.00  0.00
ATOM     86 1HG1 VAL     5      26.438 -10.605  12.238  1.00  0.00
ATOM     87 2HG1 VAL     5      27.014  -9.859  10.728  1.00  0.00
ATOM     88 3HG1 VAL     5      25.736  -9.067  11.682  1.00  0.00
ATOM     89 1HG2 VAL     5      24.209 -11.657  12.339  1.00  0.00
ATOM     90 2HG2 VAL     5      23.427 -10.156  11.786  1.00  0.00
ATOM     91 3HG2 VAL     5      23.157 -11.679  10.904  1.00  0.00
ATOM     92  N   ILE     6      25.984  -8.262   8.331  1.00  0.00
ATOM     93  CA  ILE     6      27.032  -7.710   7.507  1.00  0.00
ATOM     94  C   ILE     6      28.010  -6.961   8.387  1.00  0.00
ATOM     95  O   ILE     6      27.615  -6.359   9.380  1.00  0.00
ATOM     96  CB  ILE     6      26.465  -6.775   6.423  1.00  0.00
ATOM     97  CG1 ILE     6      25.526  -7.545   5.491  1.00  0.00
ATOM     98  CG2 ILE     6      27.594  -6.131   5.633  1.00  0.00
ATOM     99  CD1 ILE     6      24.745  -6.662   4.545  1.00  0.00
ATOM    100  H   ILE     6      25.371  -7.635   8.832  1.00  0.00
ATOM    101  HA  ILE     6      27.608  -8.505   7.035  1.00  0.00
ATOM    102  HB  ILE     6      25.867  -6.000   6.900  1.00  0.00
ATOM    103 1HG2 ILE     6      27.176  -5.474   4.871  1.00  0.00
ATOM    104 2HG2 ILE     6      28.224  -5.551   6.306  1.00  0.00
ATOM    105 3HG2 ILE     6      28.192  -6.907   5.154  1.00  0.00
ATOM    106 1HG1 ILE     6      26.137  -8.241   4.917  1.00  0.00
ATOM    107 2HG1 ILE     6      24.833  -8.105   6.120  1.00  0.00
ATOM    108 1HD1 ILE     6      24.102  -7.279   3.917  1.00  0.00
ATOM    109 2HD1 ILE     6      24.132  -5.966   5.118  1.00  0.00
ATOM    110 3HD1 ILE     6      25.435  -6.103   3.915  1.00  0.00
ATOM    111  N   VAL     7      29.290  -7.015   8.036  1.00  0.00
ATOM    112  CA  VAL     7      30.317  -6.332   8.807  1.00  0.00
```

ATOM	113	C	VAL	7	31.213	-5.481	7.909	1.00	0.00
ATOM	114	O	VAL	7	32.223	-5.968	7.402	1.00	0.00
ATOM	115	CB	VAL	7	31.193	-7.329	9.589	1.00	0.00
ATOM	116	CG1	VAL	7	32.239	-6.588	10.410	1.00	0.00
ATOM	117	CG2	VAL	7	30.332	-8.202	10.490	1.00	0.00
ATOM	118	H	VAL	7	29.556	-7.542	7.216	1.00	0.00
ATOM	119	HA	VAL	7	29.879	-5.625	9.513	1.00	0.00
ATOM	120	HB	VAL	7	31.690	-7.994	8.882	1.00	0.00
ATOM	121	1HG1	VAL	7	32.850	-7.307	10.956	1.00	0.00
ATOM	122	2HG1	VAL	7	32.875	-6.003	9.746	1.00	0.00
ATOM	123	3HG1	VAL	7	31.743	-5.924	11.117	1.00	0.00
ATOM	124	1HG2	VAL	7	30.966	-8.901	11.034	1.00	0.00
ATOM	125	2HG2	VAL	7	29.793	-7.572	11.198	1.00	0.00
ATOM	126	3HG2	VAL	7	29.618	-8.759	9.883	1.00	0.00
ATOM	127	N	PRO	8	30.822	-4.221	7.650	1.00	0.00
ATOM	128	CA	PRO	8	31.645	-3.348	6.806	1.00	0.00
ATOM	129	C	PRO	8	32.973	-3.103	7.545	1.00	0.00
ATOM	130	O	PRO	8	32.998	-2.993	8.782	1.00	0.00
ATOM	131	CB	PRO	8	30.897	-2.048	6.559	1.00	0.00
ATOM	132	CG	PRO	8	29.955	-1.910	7.726	1.00	0.00
ATOM	133	CD	PRO	8	29.555	-3.565	7.980	1.00	0.00
ATOM	134	1HD	PRO	8	29.408	-3.791	8.894	1.00	0.00
ATOM	135	2HD	PRO	8	28.955	-3.922	7.333	1.00	0.00
ATOM	136	1HG	PRO	8	30.408	-1.726	8.417	1.00	0.00
ATOM	137	2HG	PRO	8	29.146	-1.570	7.396	1.00	0.00
ATOM	138	1HB	PRO	8	31.567	-1.439	6.689	1.00	0.00
ATOM	139	2HB	PRO	8	30.424	-2.257	5.864	1.00	0.00
ATOM	140	HA	PRO	8	31.816	-3.784	5.927	1.00	0.00
ATOM	141	N	THR	9	34.078	-3.059	6.809	1.00	0.00
ATOM	142	CA	THR	9	35.380	-2.841	7.431	1.00	0.00
ATOM	143	C	THR	9	35.996	-1.468	7.194	1.00	0.00
ATOM	144	O	THR	9	37.013	-1.121	7.796	1.00	0.00
ATOM	145	CB	THR	9	36.414	-3.878	6.956	1.00	0.00
ATOM	146	OG1	THR	9	36.568	-3.787	5.533	1.00	0.00
ATOM	147	CG2	THR	9	35.967	-5.285	7.324	1.00	0.00
ATOM	148	H	THR	9	34.016	-3.178	5.808	1.00	0.00
ATOM	149	HG1	THR	9	37.214	-4.436	5.240	1.00	0.00
ATOM	150	HA	THR	9	35.287	-2.912	8.515	1.00	0.00
ATOM	151	HB	THR	9	37.372	-3.666	7.430	1.00	0.00
ATOM	152	1HG2	THR	9	36.711	-6.003	6.980	1.00	0.00
ATOM	153	2HG2	THR	9	35.860	-5.361	8.406	1.00	0.00
ATOM	154	3HG2	THR	9	35.010	-5.498	6.849	1.00	0.00
ATOM	155	N	ASN	10	35.373	-0.674	6.337	1.00	0.00
ATOM	156	CA	ASN	10	35.882	0.654	6.051	1.00	0.00
ATOM	157	C	ASN	10	34.763	1.632	5.702	1.00	0.00
ATOM	158	O	ASN	10	33.600	1.250	5.545	1.00	0.00
ATOM	159	CB	ASN	10	36.844	0.596	4.869	1.00	0.00
ATOM	160	CG	ASN	10	36.145	0.089	3.617	1.00	0.00
ATOM	161	OD1	ASN	10	35.266	0.759	3.077	1.00	0.00
ATOM	162	ND2	ASN	10	36.536	-1.101	3.155	1.00	0.00
ATOM	163	H	ASN	10	34.534	-0.997	5.877	1.00	0.00
ATOM	164	1HD2	ASN	10	36.110	-1.482	2.334	1.00	0.00
ATOM	165	2HD2	ASN	10	37.255	-1.609	3.628	1.00	0.00
ATOM	166	HA	ASN	10	36.373	1.061	6.936	1.00	0.00
ATOM	167	1HB	ASN	10	37.497	1.439	4.639	1.00	0.00
ATOM	168	2HB	ASN	10	37.432	-0.211	5.306	1.00	0.00
ATOM	169	N	GLU	11	35.141	2.887	5.514	1.00	0.00

```
ATOM    170  CA  GLU    11      34.198   3.938   5.189  1.00  0.00
ATOM    171  C   GLU    11      33.384   3.731   3.915  1.00  0.00
ATOM    172  O   GLU    11      32.183   4.015   3.898  1.00  0.00
ATOM    173  CB  GLU    11      34.917   5.283   5.063  1.00  0.00
ATOM    174  CG  GLU    11      35.438   5.843   6.379  1.00  0.00
ATOM    175  CD  GLU    11      36.154   7.149   6.172  1.00  0.00
ATOM    176  OE1 GLU    11      36.265   7.571   5.046  1.00  0.00
ATOM    177  OE2 GLU    11      36.491   7.780   7.146  1.00  0.00
ATOM    178  H   GLU    11      36.120   3.120   5.600  1.00  0.00
ATOM    179  HA  GLU    11      33.447   4.017   5.976  1.00  0.00
ATOM    180 1HB  GLU    11      35.751   5.135   4.377  1.00  0.00
ATOM    181 2HB  GLU    11      34.206   5.985   4.626  1.00  0.00
ATOM    182 1HG  GLU    11      34.662   5.971   7.133  1.00  0.00
ATOM    183 2HG  GLU    11      36.149   5.089   6.715  1.00  0.00
ATOM    184  N   GLU    12      34.024   3.235   2.857  1.00  0.00
ATOM    185  CA  GLU    12      33.330   3.014   1.591  1.00  0.00
ATOM    186  C   GLU    12      32.258   1.939   1.650  1.00  0.00
ATOM    187  O   GLU    12      31.179   2.103   1.081  1.00  0.00
ATOM    188  CB  GLU    12      34.327   2.645   0.491  1.00  0.00
ATOM    189  CG  GLU    12      35.242   3.784   0.063  1.00  0.00
ATOM    190  CD  GLU    12      36.209   3.339  -0.999  1.00  0.00
ATOM    191  OE1 GLU    12      36.183   2.185  -1.353  1.00  0.00
ATOM    192  OE2 GLU    12      36.896   4.176  -1.537  1.00  0.00
ATOM    193  H   GLU    12      35.005   3.008   2.932  1.00  0.00
ATOM    194  HA  GLU    12      32.804   3.922   1.294  1.00  0.00
ATOM    195 1HB  GLU    12      34.929   1.820   0.871  1.00  0.00
ATOM    196 2HB  GLU    12      33.745   2.306  -0.366  1.00  0.00
ATOM    197 1HG  GLU    12      34.704   4.666  -0.284  1.00  0.00
ATOM    198 2HG  GLU    12      35.789   4.029   0.972  1.00  0.00
ATOM    199  N   GLN    13      32.533   0.848   2.352  1.00  0.00
ATOM    200  CA  GLN    13      31.553  -0.214   2.456  1.00  0.00
ATOM    201  C   GLN    13      30.327   0.338   3.197  1.00  0.00
ATOM    202  O   GLN    13      29.189   0.030   2.846  1.00  0.00
ATOM    203  CB  GLN    13      32.142  -1.391   3.227  1.00  0.00
ATOM    204  CG  GLN    13      33.208  -2.073   2.378  1.00  0.00
ATOM    205  CD  GLN    13      33.641  -3.394   2.999  1.00  0.00
ATOM    206  OE1 GLN    13      33.145  -3.778   4.056  1.00  0.00
ATOM    207  NE2 GLN    13      34.570  -4.089   2.339  1.00  0.00
ATOM    208  H   GLN    13      33.426   0.757   2.814  1.00  0.00
ATOM    209 1HE2 GLN    13      34.892  -4.964   2.702  1.00  0.00
ATOM    210 2HE2 GLN    13      34.944  -3.735   1.482  1.00  0.00
ATOM    211  HA  GLN    13      31.236  -0.517   1.458  1.00  0.00
ATOM    212 1HB  GLN    13      32.552  -0.987   4.153  1.00  0.00
ATOM    213 2HB  GLN    13      31.314  -2.061   3.459  1.00  0.00
ATOM    214 1HG  GLN    13      33.109  -2.205   1.301  1.00  0.00
ATOM    215 2HG  GLN    13      33.973  -1.323   2.581  1.00  0.00
ATOM    216  N   VAL    14      30.560   1.211   4.175  1.00  0.00
ATOM    217  CA  VAL    14      29.463   1.803   4.934  1.00  0.00
ATOM    218  C   VAL    14      28.625   2.690   4.021  1.00  0.00
ATOM    219  O   VAL    14      27.393   2.647   4.052  1.00  0.00
ATOM    220  CB  VAL    14      29.981   2.624   6.130  1.00  0.00
ATOM    221  CG1 VAL    14      28.838   3.373   6.798  1.00  0.00
ATOM    222  CG2 VAL    14      30.682   1.720   7.133  1.00  0.00
ATOM    223  H   VAL    14      31.512   1.468   4.395  1.00  0.00
ATOM    224  HA  VAL    14      28.781   1.038   5.307  1.00  0.00
ATOM    225  HB  VAL    14      30.724   3.338   5.774  1.00  0.00
ATOM    226 1HG1 VAL    14      29.223   3.948   7.641  1.00  0.00
```

```
ATOM    227 2HG1 VAL    14      28.377   4.049   6.079  1.00  0.00
ATOM    228 3HG1 VAL    14      28.095   2.660   7.155  1.00  0.00
ATOM    229 1HG2 VAL    14      31.042   2.316   7.971  1.00  0.00
ATOM    230 2HG2 VAL    14      29.981   0.969   7.496  1.00  0.00
ATOM    231 3HG2 VAL    14      31.526   1.226   6.651  1.00  0.00
ATOM    232  N   ALA    15      29.300   3.507   3.219  1.00  0.00
ATOM    233  CA  ALA    15      28.613   4.399   2.302  1.00  0.00
ATOM    234  C   ALA    15      27.731   3.560   1.369  1.00  0.00
ATOM    235  O   ALA    15      26.602   3.955   1.046  1.00  0.00
ATOM    236  CB  ALA    15      29.618   5.240   1.528  1.00  0.00
ATOM    237  H   ALA    15      30.310   3.507   3.248  1.00  0.00
ATOM    238  HA  ALA    15      27.972   5.066   2.878  1.00  0.00
ATOM    239 1HB  ALA    15      29.087   5.903   0.845  1.00  0.00
ATOM    240 2HB  ALA    15      30.209   5.835   2.225  1.00  0.00
ATOM    241 3HB  ALA    15      30.277   4.586   0.959  1.00  0.00
ATOM    242  N   PHE    16      28.240   2.390   0.975  1.00  0.00
ATOM    243  CA  PHE    16      27.517   1.480   0.091  1.00  0.00
ATOM    244  C   PHE    16      26.279   0.912   0.777  1.00  0.00
ATOM    245  O   PHE    16      25.233   0.751   0.154  1.00  0.00
ATOM    246  CB  PHE    16      28.431   0.343  -0.370  1.00  0.00
ATOM    247  CG  PHE    16      27.768  -0.627  -1.305  1.00  0.00
ATOM    248  CD1 PHE    16      27.773  -0.407  -2.674  1.00  0.00
ATOM    249  CD2 PHE    16      27.137  -1.762  -0.817  1.00  0.00
ATOM    250  CE1 PHE    16      27.162  -1.299  -3.535  1.00  0.00
ATOM    251  CE2 PHE    16      26.528  -2.656  -1.676  1.00  0.00
ATOM    252  CZ  PHE    16      26.540  -2.424  -3.036  1.00  0.00
ATOM    253  H   PHE    16      29.159   2.125   1.300  1.00  0.00
ATOM    254  HD1 PHE    16      28.266   0.482  -3.068  1.00  0.00
ATOM    255  HE1 PHE    16      26.037  -3.545  -1.281  1.00  0.00
ATOM    256  HZ  PHE    16      26.058  -3.127  -3.714  1.00  0.00
ATOM    257  HE2 PHE    16      26.037  -3.545  -1.281  1.00  0.00
ATOM    258  HD2 PHE    16      27.127  -1.945   0.258  1.00  0.00
ATOM    259  HA  PHE    16      27.164   2.021  -0.788  1.00  0.00
ATOM    260 1HB  PHE    16      29.293   0.746  -0.899  1.00  0.00
ATOM    261 2HB  PHE    16      28.771  -0.235   0.488  1.00  0.00
ATOM    262  N   LEU    17      26.398   0.584   2.057  1.00  0.00
ATOM    263  CA  LEU    17      25.265   0.036   2.789  1.00  0.00
ATOM    264  C   LEU    17      24.201   1.092   3.059  1.00  0.00
ATOM    265  O   LEU    17      23.011   0.796   3.029  1.00  0.00
ATOM    266  CB  LEU    17      25.718  -0.586   4.116  1.00  0.00
ATOM    267  CG  LEU    17      26.680  -1.773   4.030  1.00  0.00
ATOM    268  CD1 LEU    17      27.166  -2.271   5.382  1.00  0.00
ATOM    269  CD2 LEU    17      26.157  -2.927   3.185  1.00  0.00
ATOM    270  H   LEU    17      27.282   0.716   2.528  1.00  0.00
ATOM    271  HA  LEU    17      24.782  -0.735   2.190  1.00  0.00
ATOM    272 1HB  LEU    17      26.236   0.270   4.545  1.00  0.00
ATOM    273 2HB  LEU    17      24.870  -0.852   4.747  1.00  0.00
ATOM    274  HG  LEU    17      27.535  -1.455   3.432  1.00  0.00
ATOM    275 1HD1 LEU    17      27.844  -3.113   5.238  1.00  0.00
ATOM    276 2HD1 LEU    17      27.692  -1.468   5.898  1.00  0.00
ATOM    277 3HD1 LEU    17      26.313  -2.590   5.980  1.00  0.00
ATOM    278 1HD2 LEU    17      26.894  -3.730   3.171  1.00  0.00
ATOM    279 2HD2 LEU    17      25.224  -3.297   3.611  1.00  0.00
ATOM    280 3HD2 LEU    17      25.979  -2.581   2.167  1.00  0.00
ATOM    281  N   GLU    18      24.628   2.326   3.303  1.00  0.00
ATOM    282  CA  GLU    18      23.697   3.412   3.567  1.00  0.00
ATOM    283  C   GLU    18      22.850   3.700   2.320  1.00  0.00
```

ATOM	284	O	GLU	18	21.669	4.060	2.418	1.00	0.00
ATOM	285	CB	GLU	18	24.451	4.669	4.008	1.00	0.00
ATOM	286	CG	GLU	18	25.124	4.553	5.369	1.00	0.00
ATOM	287	CD	GLU	18	25.864	5.813	5.721	1.00	0.00
ATOM	288	OE1	GLU	18	25.877	6.716	4.919	1.00	0.00
ATOM	289	OE2	GLU	18	26.322	5.917	6.834	1.00	0.00
ATOM	290	H	GLU	18	25.620	2.514	3.305	1.00	0.00
ATOM	291	HA	GLU	18	23.007	3.123	4.361	1.00	0.00
ATOM	292	1HB	GLU	18	25.205	4.871	3.247	1.00	0.00
ATOM	293	2HB	GLU	18	23.727	5.484	4.029	1.00	0.00
ATOM	294	1HG	GLU	18	24.434	4.301	6.174	1.00	0.00
ATOM	295	2HG	GLU	18	25.836	3.740	5.234	1.00	0.00
ATOM	296	N	ALA	19	23.463	3.531	1.147	1.00	0.00
ATOM	297	CA	ALA	19	22.782	3.755	-0.124	1.00	0.00
ATOM	298	C	ALA	19	21.703	2.696	-0.256	1.00	0.00
ATOM	299	O	ALA	19	20.563	3.015	-0.557	1.00	0.00
ATOM	300	CB	ALA	19	23.779	3.717	-1.273	1.00	0.00
ATOM	301	H	ALA	19	24.430	3.239	1.139	1.00	0.00
ATOM	302	HA	ALA	19	22.315	4.740	-0.101	1.00	0.00
ATOM	303	1HB	ALA	19	23.255	3.886	-2.214	1.00	0.00
ATOM	304	2HB	ALA	19	24.529	4.495	-1.131	1.00	0.00
ATOM	305	3HB	ALA	19	24.266	2.744	-1.300	1.00	0.00
ATOM	306	N	LEU	20	22.071	1.436	-0.036	1.00	0.00
ATOM	307	CA	LEU	20	21.119	0.333	-0.126	1.00	0.00
ATOM	308	C	LEU	20	19.922	0.620	0.781	1.00	0.00
ATOM	309	O	LEU	20	18.771	0.497	0.367	1.00	0.00
ATOM	310	CB	LEU	20	21.783	-0.998	0.252	1.00	0.00
ATOM	311	CG	LEU	20	22.897	-1.500	-0.671	1.00	0.00
ATOM	312	CD1	LEU	20	23.745	-2.607	-0.065	1.00	0.00
ATOM	313	CD2	LEU	20	22.413	-1.889	-2.061	1.00	0.00
ATOM	314	H	LEU	20	23.033	1.238	0.199	1.00	0.00
ATOM	315	HA	LEU	20	20.743	0.257	-1.146	1.00	0.00
ATOM	316	1HB	LEU	20	22.208	-0.717	1.214	1.00	0.00
ATOM	317	2HB	LEU	20	21.048	-1.789	0.397	1.00	0.00
ATOM	318	HG	LEU	20	23.531	-0.645	-0.908	1.00	0.00
ATOM	319	1HD1	LEU	20	24.512	-2.910	-0.778	1.00	0.00
ATOM	320	2HD1	LEU	20	24.221	-2.244	0.846	1.00	0.00
ATOM	321	3HD1	LEU	20	23.112	-3.462	0.171	1.00	0.00
ATOM	322	1HD2	LEU	20	23.259	-2.234	-2.657	1.00	0.00
ATOM	323	2HD2	LEU	20	21.676	-2.688	-1.980	1.00	0.00
ATOM	324	3HD2	LEU	20	21.958	-1.024	-2.544	1.00	0.00
ATOM	325	N	ALA	21	20.210	1.000	2.023	1.00	0.00
ATOM	326	CA	ALA	21	19.175	1.309	2.998	1.00	0.00
ATOM	327	C	ALA	21	18.305	2.446	2.504	1.00	0.00
ATOM	328	O	ALA	21	17.087	2.432	2.702	1.00	0.00
ATOM	329	CB	ALA	21	19.799	1.648	4.344	1.00	0.00
ATOM	330	H	ALA	21	21.179	1.077	2.298	1.00	0.00
ATOM	331	HA	ALA	21	18.538	0.433	3.121	1.00	0.00
ATOM	332	1HB	ALA	21	19.011	1.876	5.062	1.00	0.00
ATOM	333	2HB	ALA	21	20.379	0.797	4.701	1.00	0.00
ATOM	334	3HB	ALA	21	20.451	2.513	4.235	1.00	0.00
ATOM	335	N	LYS	22	18.928	3.438	1.873	1.00	0.00
ATOM	336	CA	LYS	22	18.187	4.581	1.356	1.00	0.00
ATOM	337	C	LYS	22	17.347	4.234	0.123	1.00	0.00
ATOM	338	O	LYS	22	16.374	4.922	-0.156	1.00	0.00
ATOM	339	CB	LYS	22	19.141	5.726	1.011	1.00	0.00
ATOM	340	CG	LYS	22	19.792	6.387	2.218	1.00	0.00

```
ATOM    341  CD  LYS    22      20.711   7.524   1.797  1.00  0.00
ATOM    342  CE  LYS    22      21.359   8.189   3.003  1.00  0.00
ATOM    343  NZ  LYS    22      22.280   9.288   2.604  1.00  0.00
ATOM    344  H   LYS    22      19.930   3.398   1.751  1.00  0.00
ATOM    345 1HZ  LYS    22      22.688   9.702   3.431  1.00  0.00
ATOM    346 2HZ  LYS    22      23.013   8.918   2.016  1.00  0.00
ATOM    347 3HZ  LYS    22      21.763   9.992   2.096  1.00  0.00
ATOM    348  HA  LYS    22      17.479   4.932   2.107  1.00  0.00
ATOM    349 1HB  LYS    22      19.914   5.313   0.363  1.00  0.00
ATOM    350 2HB  LYS    22      18.564   6.468   0.459  1.00  0.00
ATOM    351 1HG  LYS    22      19.006   6.775   2.866  1.00  0.00
ATOM    352 2HG  LYS    22      20.367   5.635   2.757  1.00  0.00
ATOM    353 1HD  LYS    22      21.486   7.121   1.144  1.00  0.00
ATOM    354 2HD  LYS    22      20.124   8.261   1.249  1.00  0.00
ATOM    355 1HE  LYS    22      20.570   8.589   3.638  1.00  0.00
ATOM    356 2HE  LYS    22      21.916   7.430   3.553  1.00  0.00
ATOM    357  N   GLN    23      17.707   3.170  -0.602  1.00  0.00
ATOM    358  CA  GLN    23      16.970   2.745  -1.802  1.00  0.00
ATOM    359  C   GLN    23      15.675   2.035  -1.453  1.00  0.00
ATOM    360  O   GLN    23      15.576   0.808  -1.471  1.00  0.00
ATOM    361  CB  GLN    23      17.825   1.788  -2.625  1.00  0.00
ATOM    362  CG  GLN    23      18.938   2.567  -3.318  1.00  0.00
ATOM    363  CD  GLN    23      18.370   3.538  -4.344  1.00  0.00
ATOM    364  OE1 GLN    23      18.645   4.734  -4.289  1.00  0.00
ATOM    365  NE2 GLN    23      17.573   3.019  -5.281  1.00  0.00
ATOM    366  H   GLN    23      18.516   2.638  -0.315  1.00  0.00
ATOM    367 1HE2 GLN    23      17.172   3.612  -5.980  1.00  0.00
ATOM    368 2HE2 GLN    23      17.377   2.038  -5.285  1.00  0.00
ATOM    369  HA  GLN    23      16.687   3.621  -2.386  1.00  0.00
ATOM    370 1HB  GLN    23      18.217   1.042  -1.934  1.00  0.00
ATOM    371 2HB  GLN    23      17.158   1.306  -3.340  1.00  0.00
ATOM    372 1HG  GLN    23      19.741   3.061  -2.771  1.00  0.00
ATOM    373 2HG  GLN    23      19.338   1.698  -3.842  1.00  0.00
ATOM    374  N   ASP    24      14.674   2.844  -1.169  1.00  0.00
ATOM    375  CA  ASP    24      13.347   2.393  -0.808  1.00  0.00
ATOM    376  C   ASP    24      12.743   1.308  -1.709  1.00  0.00
ATOM    377  O   ASP    24      12.108   0.369  -1.224  1.00  0.00
ATOM    378  CB  ASP    24      12.369   3.570  -0.783  1.00  0.00
ATOM    379  CG  ASP    24      12.562   4.524   0.388  1.00  0.00
ATOM    380  OD1 ASP    24      13.293   4.187   1.289  1.00  0.00
ATOM    381  OD2 ASP    24      12.108   5.640   0.300  1.00  0.00
ATOM    382  H   ASP    24      14.843   3.839  -1.206  1.00  0.00
ATOM    383  HA  ASP    24      13.369   1.937   0.182  1.00  0.00
ATOM    384 1HB  ASP    24      12.349   4.138  -1.714  1.00  0.00
ATOM    385 2HB  ASP    24      11.427   3.034  -0.663  1.00  0.00
ATOM    386  N   GLU    25      13.010   1.383  -3.009  1.00  0.00
ATOM    387  CA  GLU    25      12.467   0.400  -3.941  1.00  0.00
ATOM    388  C   GLU    25      13.031  -1.010  -3.893  1.00  0.00
ATOM    389  O   GLU    25      12.450  -1.928  -4.470  1.00  0.00
ATOM    390  CB  GLU    25      12.631   0.882  -5.384  1.00  0.00
ATOM    391  CG  GLU    25      11.857   2.151  -5.715  1.00  0.00
ATOM    392  CD  GLU    25      12.088   2.576  -7.138  1.00  0.00
ATOM    393  OE1 GLU    25      12.846   1.926  -7.817  1.00  0.00
ATOM    394  OE2 GLU    25      11.422   3.482  -7.582  1.00  0.00
ATOM    395  H   GLU    25      13.596   2.129  -3.356  1.00  0.00
ATOM    396  HA  GLU    25      11.405   0.249  -3.743  1.00  0.00
ATOM    397 1HB  GLU    25      13.696   1.055  -5.543  1.00  0.00
```

```
ATOM    398 2HB  GLU    25      12.294   0.072  -6.031  1.00  0.00
ATOM    399 1HG  GLU    25      10.786   2.066  -5.532  1.00  0.00
ATOM    400 2HG  GLU    25      12.282   2.893  -5.041  1.00  0.00
ATOM    401  N   LEU    26      14.154  -1.186  -3.203  1.00  0.00
ATOM    402  CA  LEU    26      14.767  -2.502  -3.083  1.00  0.00
ATOM    403  C   LEU    26      14.031  -3.298  -1.995  1.00  0.00
ATOM    404  O   LEU    26      14.189  -4.510  -1.876  1.00  0.00
ATOM    405  CB  LEU    26      16.263  -2.384  -2.763  1.00  0.00
ATOM    406  CG  LEU    26      17.196  -2.067  -3.935  1.00  0.00
ATOM    407  CD1 LEU    26      18.648  -1.863  -3.527  1.00  0.00
ATOM    408  CD2 LEU    26      17.093  -3.057  -5.086  1.00  0.00
ATOM    409  H   LEU    26      14.590  -0.393  -2.754  1.00  0.00
ATOM    410  HA  LEU    26      14.653  -3.045  -4.021  1.00  0.00
ATOM    411 1HB  LEU    26      16.232  -1.534  -2.083  1.00  0.00
ATOM    412 2HB  LEU    26      16.633  -3.261  -2.232  1.00  0.00
ATOM    413  HG  LEU    26      16.822  -1.160  -4.411  1.00  0.00
ATOM    414 1HD1 LEU    26      19.247  -1.642  -4.411  1.00  0.00
ATOM    415 2HD1 LEU    26      18.716  -1.030  -2.827  1.00  0.00
ATOM    416 3HD1 LEU    26      19.023  -2.769  -3.052  1.00  0.00
ATOM    417 1HD2 LEU    26      17.783  -2.766  -5.879  1.00  0.00
ATOM    418 2HD2 LEU    26      17.347  -4.056  -4.731  1.00  0.00
ATOM    419 3HD2 LEU    26      16.075  -3.059  -5.475  1.00  0.00
ATOM    420  N   ASN    27      13.223  -2.600  -1.205  1.00  0.00
ATOM    421  CA  ASN    27      12.449  -3.223  -0.128  1.00  0.00
ATOM    422  C   ASN    27      13.246  -3.994   0.935  1.00  0.00
ATOM    423  O   ASN    27      12.778  -5.021   1.451  1.00  0.00
ATOM    424  CB  ASN    27      11.465  -4.230  -0.713  1.00  0.00
ATOM    425  CG  ASN    27      10.383  -3.532  -1.523  1.00  0.00
ATOM    426  OD1 ASN    27       9.703  -2.640  -1.020  1.00  0.00
ATOM    427  ND2 ASN    27      10.223  -3.941  -2.784  1.00  0.00
ATOM    428  H   ASN    27      13.139  -1.604  -1.352  1.00  0.00
ATOM    429 1HD2 ASN    27       9.526  -3.517  -3.362  1.00  0.00
ATOM    430 2HD2 ASN    27      10.800  -4.671  -3.150  1.00  0.00
ATOM    431  HA  ASN    27      11.928  -2.455   0.446  1.00  0.00
ATOM    432 1HB  ASN    27      11.820  -5.126  -1.223  1.00  0.00
ATOM    433 2HB  ASN    27      11.065  -4.503   0.264  1.00  0.00
ATOM    434  N   PHE    28      14.455  -3.531   1.236  1.00  0.00
ATOM    435  CA  PHE    28      15.275  -4.192   2.245  1.00  0.00
ATOM    436  C   PHE    28      14.734  -3.664   3.584  1.00  0.00
ATOM    437  O   PHE    28      14.174  -2.550   3.646  1.00  0.00
ATOM    438  CB  PHE    28      16.756  -3.883   2.016  1.00  0.00
ATOM    439  CG  PHE    28      17.267  -4.326   0.675  1.00  0.00
ATOM    440  CD1 PHE    28      16.536  -5.211  -0.104  1.00  0.00
ATOM    441  CD2 PHE    28      18.479  -3.859   0.189  1.00  0.00
ATOM    442  CE1 PHE    28      17.006  -5.619  -1.338  1.00  0.00
ATOM    443  CE2 PHE    28      18.950  -4.265  -1.045  1.00  0.00
ATOM    444  CZ  PHE    28      18.212  -5.146  -1.809  1.00  0.00
ATOM    445  H   PHE    28      14.810  -2.713   0.761  1.00  0.00
ATOM    446  HD1 PHE    28      15.582  -5.585   0.267  1.00  0.00
ATOM    447  HE1 PHE    28      19.904  -3.891  -1.415  1.00  0.00
ATOM    448  HZ  PHE    28      18.583  -5.468  -2.781  1.00  0.00
ATOM    449  HE2 PHE    28      19.904  -3.891  -1.415  1.00  0.00
ATOM    450  HD2 PHE    28      19.062  -3.163   0.793  1.00  0.00
ATOM    451  HA  PHE    28      15.132  -5.272   2.189  1.00  0.00
ATOM    452 1HB  PHE    28      16.929  -2.809   2.076  1.00  0.00
ATOM    453 2HB  PHE    28      17.364  -4.390   2.764  1.00  0.00
ATOM    454  N   ASP    29      14.900  -4.454   4.641  1.00  0.00
```

```
ATOM    455  CA  ASP    29      14.427  -4.083   5.971  1.00  0.00
ATOM    456  C   ASP    29      15.614  -4.181   6.928  1.00  0.00
ATOM    457  O   ASP    29      16.009  -5.267   7.338  1.00  0.00
ATOM    458  CB  ASP    29      13.269  -4.987   6.400  1.00  0.00
ATOM    459  CG  ASP    29      12.465  -4.461   7.581  1.00  0.00
ATOM    460  OD1 ASP    29      12.812  -3.425   8.098  1.00  0.00
ATOM    461  OD2 ASP    29      11.425  -5.010   7.858  1.00  0.00
ATOM    462  H   ASP    29      15.369  -5.340   4.519  1.00  0.00
ATOM    463  HA  ASP    29      14.078  -3.050   5.964  1.00  0.00
ATOM    464 1HB  ASP    29      12.592  -5.242   5.585  1.00  0.00
ATOM    465 2HB  ASP    29      13.823  -5.876   6.705  1.00  0.00
ATOM    466  N   TRP    30      16.230  -3.047   7.231  1.00  0.00
ATOM    467  CA  TRP    30      17.376  -3.043   8.128  1.00  0.00
ATOM    468  C   TRP    30      16.947  -3.140   9.577  1.00  0.00
ATOM    469  O   TRP    30      16.316  -2.233  10.108  1.00  0.00
ATOM    470  CB  TRP    30      18.214  -1.781   7.917  1.00  0.00
ATOM    471  CG  TRP    30      18.879  -1.722   6.575  1.00  0.00
ATOM    472  CD1 TRP    30      18.344  -1.235   5.420  1.00  0.00
ATOM    473  CD2 TRP    30      20.203  -2.164   6.250  1.00  0.00
ATOM    474  NE1 TRP    30      19.250  -1.347   4.395  1.00  0.00
ATOM    475  CE2 TRP    30      20.401  -1.914   4.880  1.00  0.00
ATOM    476  CE3 TRP    30      21.241  -2.747   6.987  1.00  0.00
ATOM    477  CZ2 TRP    30      21.586  -2.225   4.232  1.00  0.00
ATOM    478  CZ3 TRP    30      22.429  -3.059   6.337  1.00  0.00
ATOM    479  CH2 TRP    30      22.597  -2.804   4.999  1.00  0.00
ATOM    480  H   TRP    30      15.901  -2.178   6.836  1.00  0.00
ATOM    481  HE1 TRP    30      19.095  -1.060   3.439  1.00  0.00
ATOM    482  HD1 TRP    30      17.328  -0.847   5.474  1.00  0.00
ATOM    483  HZ2 TRP    30      21.676  -2.007   3.167  1.00  0.00
ATOM    484  HH2 TRP    30      23.545  -3.064   4.527  1.00  0.00
ATOM    485  HZ3 TRP    30      23.229  -3.513   6.922  1.00  0.00
ATOM    486  HE3 TRP    30      21.162  -2.968   8.051  1.00  0.00
ATOM    487  HA  TRP    30      18.002  -3.913   7.932  1.00  0.00
ATOM    488 1HB  TRP    30      17.586  -0.893   7.993  1.00  0.00
ATOM    489 2HB  TRP    30      19.008  -1.729   8.662  1.00  0.00
ATOM    490  N   GLN    31      17.235  -4.280  10.183  1.00  0.00
ATOM    491  CA  GLN    31      16.894  -4.520  11.574  1.00  0.00
ATOM    492  C   GLN    31      17.869  -3.739  12.450  1.00  0.00
ATOM    493  O   GLN    31      17.507  -3.240  13.519  1.00  0.00
ATOM    494  CB  GLN    31      16.997  -6.009  11.885  1.00  0.00
ATOM    495  CG  GLN    31      16.157  -6.797  10.885  1.00  0.00
ATOM    496  CD  GLN    31      14.676  -6.483  11.045  1.00  0.00
ATOM    497  OE1 GLN    31      14.124  -6.622  12.135  1.00  0.00
ATOM    498  NE2 GLN    31      14.034  -6.061   9.954  1.00  0.00
ATOM    499  H   GLN    31      17.707  -5.006   9.662  1.00  0.00
ATOM    500 1HE2 GLN    31      13.059  -5.840  10.002  1.00  0.00
ATOM    501 2HE2 GLN    31      14.526  -5.964   9.090  1.00  0.00
ATOM    502  HA  GLN    31      15.890  -4.145  11.774  1.00  0.00
ATOM    503 1HB  GLN    31      18.054  -6.270  11.828  1.00  0.00
ATOM    504 2HB  GLN    31      16.645  -6.142  12.908  1.00  0.00
ATOM    505 1HG  GLN    31      16.395  -6.844   9.823  1.00  0.00
ATOM    506 2HG  GLN    31      16.361  -7.765  11.344  1.00  0.00
ATOM    507  N   ASN    32      19.091  -3.581  11.955  1.00  0.00
ATOM    508  CA  ASN    32      20.145  -2.854  12.656  1.00  0.00
ATOM    509  C   ASN    32      20.937  -2.167  11.534  1.00  0.00
ATOM    510  O   ASN    32      21.612  -2.812  10.728  1.00  0.00
ATOM    511  CB  ASN    32      21.009  -3.830  13.448  1.00  0.00
```

```
ATOM    512  CG  ASN    32      22.043  -3.092  14.285  1.00  0.00
ATOM    513  OD1 ASN    32      22.144  -1.869  14.218  1.00  0.00
ATOM    514  ND2 ASN    32      22.813  -3.841  15.078  1.00  0.00
ATOM    515  H   ASN    32      19.301  -3.982  11.052  1.00  0.00
ATOM    516 1HD2 ASN    32      23.510  -3.407  15.651  1.00  0.00
ATOM    517 2HD2 ASN    32      22.694  -4.833  15.099  1.00  0.00
ATOM    518  HA  ASN    32      19.703  -2.102  13.311  1.00  0.00
ATOM    519 1HB  ASN    32      20.555  -4.645  14.014  1.00  0.00
ATOM    520 2HB  ASN    32      21.487  -4.231  12.554  1.00  0.00
ATOM    521  N   PRO    33      20.771  -0.858  11.447  1.00  0.00
ATOM    522  CA  PRO    33      21.431  -0.058  10.437  1.00  0.00
ATOM    523  C   PRO    33      22.949  -0.024  10.543  1.00  0.00
ATOM    524  O   PRO    33      23.514  -0.328  11.594  1.00  0.00
ATOM    525  CB  PRO    33      20.925   1.372  10.528  1.00  0.00
ATOM    526  CG  PRO    33      19.591   1.274  11.219  1.00  0.00
ATOM    527  CD  PRO    33      19.830  -0.046  12.234  1.00  0.00
ATOM    528 1HD  PRO    33      20.312   0.163  13.030  1.00  0.00
ATOM    529 2HD  PRO    33      19.070  -0.615  12.312  1.00  0.00
ATOM    530 1HG  PRO    33      19.530   1.909  11.776  1.00  0.00
ATOM    531 2HG  PRO    33      18.981   0.917  10.604  1.00  0.00
ATOM    532 1HB  PRO    33      21.511   1.721  11.138  1.00  0.00
ATOM    533 2HB  PRO    33      20.775   1.549   9.694  1.00  0.00
ATOM    534  HA  PRO    33      21.218  -0.409   9.529  1.00  0.00
ATOM    535  N   PRO    34      23.632   0.277   9.421  1.00  0.00
ATOM    536  CA  PRO    34      25.098   0.349   9.438  1.00  0.00
ATOM    537  C   PRO    34      25.475   1.604  10.210  1.00  0.00
ATOM    538  O   PRO    34      24.784   2.620  10.124  1.00  0.00
ATOM    539  CB  PRO    34      25.609   0.393   8.008  1.00  0.00
ATOM    540  CG  PRO    34      24.472   0.963   7.202  1.00  0.00
ATOM    541  CD  PRO    34      23.138   0.271   8.043  1.00  0.00
ATOM    542 1HD  PRO    34      22.366   0.830   8.070  1.00  0.00
ATOM    543 2HD  PRO    34      23.008  -0.656   7.867  1.00  0.00
ATOM    544 1HG  PRO    34      24.396   1.785   7.389  1.00  0.00
ATOM    545 2HG  PRO    34      24.443   0.498   6.389  1.00  0.00
ATOM    546 1HB  PRO    34      26.240   1.052   8.073  1.00  0.00
ATOM    547 2HB  PRO    34      25.663  -0.451   7.824  1.00  0.00
ATOM    548  HA  PRO    34      25.475  -0.459   9.881  1.00  0.00
ATOM    549  N   THR    35      26.553   1.536  10.974  1.00  0.00
ATOM    550  CA  THR    35      26.978   2.686  11.748  1.00  0.00
ATOM    551  C   THR    35      28.421   3.103  11.482  1.00  0.00
ATOM    552  O   THR    35      28.671   4.092  10.785  1.00  0.00
ATOM    553  CB  THR    35      26.835   2.434  13.260  1.00  0.00
ATOM    554  OG1 THR    35      27.542   1.239  13.617  1.00  0.00
ATOM    555  CG2 THR    35      25.369   2.283  13.639  1.00  0.00
ATOM    556  H   THR    35      27.083   0.678  11.018  1.00  0.00
ATOM    557  HG1 THR    35      27.452   1.084  14.560  1.00  0.00
ATOM    558  HA  THR    35      26.375   3.554  11.480  1.00  0.00
ATOM    559  HB  THR    35      27.266   3.276  13.802  1.00  0.00
ATOM    560 1HG2 THR    35      25.288   2.106  14.711  1.00  0.00
ATOM    561 2HG2 THR    35      24.831   3.194  13.379  1.00  0.00
ATOM    562 3HG2 THR    35      24.939   1.441  13.098  1.00  0.00
ATOM    563  N   GLU    36      29.364   2.340  12.028  1.00  0.00
ATOM    564  CA  GLU    36      30.788   2.629  11.863  1.00  0.00
ATOM    565  C   GLU    36      31.522   1.386  11.396  1.00  0.00
ATOM    566  O   GLU    36      31.022   0.260  11.544  1.00  0.00
ATOM    567  CB  GLU    36      31.385   3.149  13.173  1.00  0.00
ATOM    568  CG  GLU    36      30.777   4.454  13.666  1.00  0.00
```

```
ATOM    569  CD  GLU    36      31.393   4.886  14.968  1.00  0.00
ATOM    570  OE1 GLU    36      32.229   4.176  15.472  1.00  0.00
ATOM    571  OE2 GLU    36      31.113   5.979  15.401  1.00  0.00
ATOM    572  H   GLU    36      29.088   1.536  12.573  1.00  0.00
ATOM    573  HA  GLU    36      30.924   3.389  11.093  1.00  0.00
ATOM    574 1HB  GLU    36      31.233   2.372  13.923  1.00  0.00
ATOM    575 2HB  GLU    36      32.453   3.287  13.004  1.00  0.00
ATOM    576 1HG  GLU    36      30.850   5.266  12.943  1.00  0.00
ATOM    577 2HG  GLU    36      29.729   4.204  13.826  1.00  0.00
ATOM    578  N   PRO    37      32.712   1.573  10.796  1.00  0.00
ATOM    579  CA  PRO    37      33.478   0.415  10.327  1.00  0.00
ATOM    580  C   PRO    37      33.722  -0.540  11.492  1.00  0.00
ATOM    581  O   PRO    37      33.877  -0.104  12.632  1.00  0.00
ATOM    582  CB  PRO    37      34.797   0.895   9.746  1.00  0.00
ATOM    583  CG  PRO    37      34.556   2.330   9.358  1.00  0.00
ATOM    584  CD  PRO    37      33.457   2.811  10.556  1.00  0.00
ATOM    585 1HD  PRO    37      33.865   3.003  11.396  1.00  0.00
ATOM    586 2HD  PRO    37      32.784   3.408  10.243  1.00  0.00
ATOM    587 1HG  PRO    37      35.243   2.782   9.560  1.00  0.00
ATOM    588 2HG  PRO    37      34.006   2.328   8.599  1.00  0.00
ATOM    589 1HB  PRO    37      35.318   0.911  10.498  1.00  0.00
ATOM    590 2HB  PRO    37      34.837   0.430   9.017  1.00  0.00
ATOM    591  HA  PRO    37      32.975  -0.062   9.611  1.00  0.00
ATOM    592  N   GLY    38      33.652  -1.836  11.227  1.00  0.00
ATOM    593  CA  GLY    38      33.875  -2.805  12.277  1.00  0.00
ATOM    594  C   GLY    38      32.624  -3.235  13.010  1.00  0.00
ATOM    595  O   GLY    38      32.650  -4.259  13.700  1.00  0.00
ATOM    596  H   GLY    38      33.442  -2.148  10.290  1.00  0.00
ATOM    597 1HA  GLY    38      34.323  -3.701  11.846  1.00  0.00
ATOM    598 2HA  GLY    38      34.554  -2.380  13.015  1.00  0.00
ATOM    599  N   GLN    39      31.531  -2.479  12.867  1.00  0.00
ATOM    600  CA  GLN    39      30.263  -2.808  13.535  1.00  0.00
ATOM    601  C   GLN    39      29.292  -3.536  12.609  1.00  0.00
ATOM    602  O   GLN    39      29.153  -3.175  11.443  1.00  0.00
ATOM    603  CB  GLN    39      29.582  -1.531  14.016  1.00  0.00
ATOM    604  CG  GLN    39      30.456  -0.852  15.064  1.00  0.00
ATOM    605  CD  GLN    39      30.574  -1.708  16.318  1.00  0.00
ATOM    606  OE1 GLN    39      29.567  -2.086  16.911  1.00  0.00
ATOM    607  NE2 GLN    39      31.811  -2.010  16.720  1.00  0.00
ATOM    608  H   GLN    39      31.577  -1.657  12.282  1.00  0.00
ATOM    609 1HE2 GLN    39      31.946  -2.569  17.539  1.00  0.00
ATOM    610 2HE2 GLN    39      32.600  -1.679  16.204  1.00  0.00
ATOM    611  HA  GLN    39      30.453  -3.488  14.365  1.00  0.00
ATOM    612 1HB  GLN    39      29.433  -0.903  13.137  1.00  0.00
ATOM    613 2HB  GLN    39      28.613  -1.824  14.420  1.00  0.00
ATOM    614 1HG  GLN    39      31.433  -0.429  14.833  1.00  0.00
ATOM    615 2HG  GLN    39      29.749  -0.044  15.257  1.00  0.00
ATOM    616  N   PRO    40      28.619  -4.551  13.143  1.00  0.00
ATOM    617  CA  PRO    40      27.662  -5.329  12.366  1.00  0.00
ATOM    618  C   PRO    40      26.360  -4.587  12.048  1.00  0.00
ATOM    619  O   PRO    40      25.974  -3.645  12.751  1.00  0.00
ATOM    620  CB  PRO    40      27.311  -6.593  13.133  1.00  0.00
ATOM    621  CG  PRO    40      27.552  -6.253  14.581  1.00  0.00
ATOM    622  CD  PRO    40      28.936  -5.228  14.405  1.00  0.00
ATOM    623 1HD  PRO    40      28.980  -4.521  15.043  1.00  0.00
ATOM    624 2HD  PRO    40      29.742  -5.693  14.201  1.00  0.00
ATOM    625 1HG  PRO    40      26.953  -5.710  14.833  1.00  0.00
```

```
ATOM    626 2HG  PRO    40      27.926  -7.009  14.989  1.00  0.00
ATOM    627 1HB  PRO    40      26.404  -6.616  13.017  1.00  0.00
ATOM    628 2HB  PRO    40      27.946  -7.124  12.879  1.00  0.00
ATOM    629  HA  PRO    40      28.064  -5.593  11.493  1.00  0.00
ATOM    630  N   VAL    41      25.718  -4.998  10.958  1.00  0.00
ATOM    631  CA  VAL    41      24.463  -4.422  10.503  1.00  0.00
ATOM    632  C   VAL    41      23.629  -5.655  10.225  1.00  0.00
ATOM    633  O   VAL    41      24.172  -6.707   9.904  1.00  0.00
ATOM    634  CB  VAL    41      24.669  -3.518   9.273  1.00  0.00
ATOM    635  CG1 VAL    41      25.803  -2.534   9.519  1.00  0.00
ATOM    636  CG2 VAL    41      24.953  -4.357   8.037  1.00  0.00
ATOM    637  H   VAL    41      26.123  -5.749  10.418  1.00  0.00
ATOM    638  HA  VAL    41      23.987  -3.835  11.290  1.00  0.00
ATOM    639  HB  VAL    41      23.747  -2.969   9.079  1.00  0.00
ATOM    640 1HG1 VAL    41      25.934  -1.903   8.640  1.00  0.00
ATOM    641 2HG1 VAL    41      25.563  -1.910  10.380  1.00  0.00
ATOM    642 3HG1 VAL    41      26.725  -3.082   9.712  1.00  0.00
ATOM    643 1HG2 VAL    41      25.095  -3.703   7.177  1.00  0.00
ATOM    644 2HG2 VAL    41      25.856  -4.947   8.198  1.00  0.00
ATOM    645 3HG2 VAL    41      24.112  -5.024   7.849  1.00  0.00
ATOM    646  N   VAL    42      22.323  -5.572  10.436  1.00  0.00
ATOM    647  CA  VAL    42      21.458  -6.721  10.194  1.00  0.00
ATOM    648  C   VAL    42      20.408  -6.257   9.218  1.00  0.00
ATOM    649  O   VAL    42      19.820  -5.190   9.421  1.00  0.00
ATOM    650  CB  VAL    42      20.833  -7.243  11.501  1.00  0.00
ATOM    651  CG1 VAL    42      19.898  -8.409  11.216  1.00  0.00
ATOM    652  CG2 VAL    42      21.919  -7.661  12.482  1.00  0.00
ATOM    653  H   VAL    42      21.923  -4.706  10.767  1.00  0.00
ATOM    654  HA  VAL    42      22.005  -7.537   9.720  1.00  0.00
ATOM    655  HB  VAL    42      20.273  -6.435  11.973  1.00  0.00
ATOM    656 1HG1 VAL    42      19.465  -8.765  12.151  1.00  0.00
ATOM    657 2HG1 VAL    42      19.101  -8.081  10.549  1.00  0.00
ATOM    658 3HG1 VAL    42      20.457  -9.217  10.745  1.00  0.00
ATOM    659 1HG2 VAL    42      21.460  -8.026  13.400  1.00  0.00
ATOM    660 2HG2 VAL    42      22.524  -8.452  12.038  1.00  0.00
ATOM    661 3HG2 VAL    42      22.553  -6.804  12.710  1.00  0.00
ATOM    662  N   ILE    43      20.143  -7.059   8.189  1.00  0.00
ATOM    663  CA  ILE    43      19.133  -6.692   7.192  1.00  0.00
ATOM    664  C   ILE    43      18.286  -7.852   6.663  1.00  0.00
ATOM    665  O   ILE    43      18.808  -8.931   6.362  1.00  0.00
ATOM    666  CB  ILE    43      19.770  -6.010   5.967  1.00  0.00
ATOM    667  CG1 ILE    43      18.686  -5.560   4.984  1.00  0.00
ATOM    668  CG2 ILE    43      20.754  -6.948   5.287  1.00  0.00
ATOM    669  CD1 ILE    43      19.230  -4.895   3.739  1.00  0.00
ATOM    670  H   ILE    43      20.644  -7.931   8.094  1.00  0.00
ATOM    671  HA  ILE    43      18.386  -6.030   7.628  1.00  0.00
ATOM    672  HB  ILE    43      20.292  -5.111   6.293  1.00  0.00
ATOM    673 1HG2 ILE    43      21.194  -6.450   4.423  1.00  0.00
ATOM    674 2HG2 ILE    43      21.541  -7.220   5.989  1.00  0.00
ATOM    675 3HG2 ILE    43      20.233  -7.848   4.960  1.00  0.00
ATOM    676 1HG1 ILE    43      18.114  -6.444   4.704  1.00  0.00
ATOM    677 2HG1 ILE    43      18.037  -4.863   5.514  1.00  0.00
ATOM    678 1HD1 ILE    43      18.403  -4.603   3.091  1.00  0.00
ATOM    679 2HD1 ILE    43      19.801  -4.009   4.019  1.00  0.00
ATOM    680 3HD1 ILE    43      19.878  -5.591   3.208  1.00  0.00
ATOM    681  N   LEU    44      16.970  -7.672   6.681  1.00  0.00
ATOM    682  CA  LEU    44      16.063  -8.693   6.180  1.00  0.00
```

```
ATOM    683  C   LEU    44      15.940  -8.404   4.674  1.00  0.00
ATOM    684  O   LEU    44      15.591  -7.293   4.266  1.00  0.00
ATOM    685  CB  LEU    44      14.719  -8.644   6.918  1.00  0.00
ATOM    686  CG  LEU    44      13.578  -9.480   6.333  1.00  0.00
ATOM    687  CD1 LEU    44      13.848 -10.977   6.339  1.00  0.00
ATOM    688  CD2 LEU    44      12.217  -9.173   6.941  1.00  0.00
ATOM    689  H   LEU    44      16.592  -6.811   7.049  1.00  0.00
ATOM    690  HA  LEU    44      16.505  -9.678   6.327  1.00  0.00
ATOM    691 1HB  LEU    44      15.026  -9.067   7.873  1.00  0.00
ATOM    692 2HB  LEU    44      14.378  -7.620   7.067  1.00  0.00
ATOM    693  HG  LEU    44      13.422  -9.143   5.308  1.00  0.00
ATOM    694 1HD1 LEU    44      12.995 -11.503   5.909  1.00  0.00
ATOM    695 2HD1 LEU    44      14.739 -11.188   5.747  1.00  0.00
ATOM    696 3HD1 LEU    44      14.003 -11.314   7.363  1.00  0.00
ATOM    697 1HD2 LEU    44      11.460  -9.805   6.475  1.00  0.00
ATOM    698 2HD2 LEU    44      12.244  -9.369   8.013  1.00  0.00
ATOM    699 3HD2 LEU    44      11.970  -8.125   6.770  1.00  0.00
ATOM    700  N   ILE    45      16.249  -9.404   3.860  1.00  0.00
ATOM    701  CA  ILE    45      16.181  -9.284   2.403  1.00  0.00
ATOM    702  C   ILE    45      15.045 -10.159   1.885  1.00  0.00
ATOM    703  O   ILE    45      15.035 -11.376   2.115  1.00  0.00
ATOM    704  CB  ILE    45      17.511  -9.687   1.740  1.00  0.00
ATOM    705  CG1 ILE    45      18.649  -8.794   2.240  1.00  0.00
ATOM    706  CG2 ILE    45      17.395  -9.610   0.226  1.00  0.00
ATOM    707  CD1 ILE    45      18.567  -7.367   1.748  1.00  0.00
ATOM    708  H   ILE    45      16.543 -10.283   4.261  1.00  0.00
ATOM    709  HA  ILE    45      15.926  -8.265   2.113  1.00  0.00
ATOM    710  HB  ILE    45      17.760 -10.706   2.034  1.00  0.00
ATOM    711 1HG2 ILE    45      18.344  -9.897  -0.227  1.00  0.00
ATOM    712 2HG2 ILE    45      16.612 -10.286  -0.114  1.00  0.00
ATOM    713 3HG2 ILE    45      17.147  -8.590  -0.069  1.00  0.00
ATOM    714 1HG1 ILE    45      18.616  -8.805   3.329  1.00  0.00
ATOM    715 2HG1 ILE    45      19.584  -9.241   1.902  1.00  0.00
ATOM    716 1HD1 ILE    45      19.407  -6.796   2.144  1.00  0.00
ATOM    717 2HD1 ILE    45      18.602  -7.354   0.658  1.00  0.00
ATOM    718 3HD1 ILE    45      17.634  -6.918   2.086  1.00  0.00
ATOM    719  N   PRO    46      14.072  -9.559   1.180  1.00  0.00
ATOM    720  CA  PRO    46      12.949 -10.344   0.650  1.00  0.00
ATOM    721  C   PRO    46      13.474 -11.491  -0.209  1.00  0.00
ATOM    722  O   PRO    46      14.459 -11.325  -0.931  1.00  0.00
ATOM    723  CB  PRO    46      12.054  -9.435  -0.175  1.00  0.00
ATOM    724  CG  PRO    46      12.326  -8.045   0.338  1.00  0.00
ATOM    725  CD  PRO    46      13.967  -8.165   0.747  1.00  0.00
ATOM    726 1HD  PRO    46      14.553  -8.110  -0.003  1.00  0.00
ATOM    727 2HD  PRO    46      14.204  -7.679   1.531  1.00  0.00
ATOM    728 1HG  PRO    46      12.349  -7.515  -0.322  1.00  0.00
ATOM    729 2HG  PRO    46      11.933  -7.976   1.186  1.00  0.00
ATOM    730 1HB  PRO    46      12.458  -9.496  -0.993  1.00  0.00
ATOM    731 2HB  PRO    46      11.271  -9.670   0.109  1.00  0.00
ATOM    732  HA  PRO    46      12.410 -10.720   1.399  1.00  0.00
ATOM    733  N   SER    47      12.804 -12.639  -0.141  1.00  0.00
ATOM    734  CA  SER    47      13.188 -13.824  -0.900  1.00  0.00
ATOM    735  C   SER    47      13.625 -13.556  -2.341  1.00  0.00
ATOM    736  O   SER    47      14.685 -13.992  -2.778  1.00  0.00
ATOM    737  CB  SER    47      11.901 -14.653  -0.918  1.00  0.00
ATOM    738  OG  SER    47      10.850 -13.949  -1.555  1.00  0.00
ATOM    739  H   SER    47      11.995 -12.692   0.462  1.00  0.00
```

```
ATOM    740  HG  SER    47       10.056 -14.489  -1.553  1.00  0.00
ATOM    741  HA  SER    47       13.974 -14.409  -0.421  1.00  0.00
ATOM    742 1HB  SER    47       12.093 -15.582  -1.454  1.00  0.00
ATOM    743 2HB  SER    47       11.617 -14.879   0.109  1.00  0.00
ATOM    744  N   ASP    48       12.772 -12.872  -3.082  1.00  0.00
ATOM    745  CA  ASP    48       13.054 -12.546  -4.467  1.00  0.00
ATOM    746  C   ASP    48       14.384 -11.815  -4.713  1.00  0.00
ATOM    747  O   ASP    48       15.036 -12.058  -5.731  1.00  0.00
ATOM    748  CB  ASP    48       11.932 -11.687  -5.055  1.00  0.00
ATOM    749  CG  ASP    48       10.625 -12.433  -5.287  1.00  0.00
ATOM    750  OD1 ASP    48       10.618 -13.634  -5.161  1.00  0.00
ATOM    751  OD2 ASP    48        9.614 -11.787  -5.432  1.00  0.00
ATOM    752  H   ASP    48       11.900 -12.568  -2.673  1.00  0.00
ATOM    753  HA  ASP    48       13.135 -13.462  -5.053  1.00  0.00
ATOM    754 1HB  ASP    48       11.735 -10.779  -4.484  1.00  0.00
ATOM    755 2HB  ASP    48       12.375 -11.424  -6.016  1.00  0.00
ATOM    756  N   MET    49       14.815 -10.982  -3.763  1.00  0.00
ATOM    757  CA  MET    49       16.069 -10.228  -3.901  1.00  0.00
ATOM    758  C   MET    49       17.268 -10.821  -3.197  1.00  0.00
ATOM    759  O   MET    49       18.338 -10.212  -3.218  1.00  0.00
ATOM    760  CB  MET    49       15.885  -8.800  -3.390  1.00  0.00
ATOM    761  CG  MET    49       14.839  -7.992  -4.144  1.00  0.00
ATOM    762  SD  MET    49       15.277  -7.729  -5.874  1.00  0.00
ATOM    763  CE  MET    49       16.669  -6.616  -5.706  1.00  0.00
ATOM    764  H   MET    49       14.263 -10.867  -2.925  1.00  0.00
ATOM    765  HA  MET    49       16.365 -10.188  -4.949  1.00  0.00
ATOM    766 1HB  MET    49       15.602  -8.873  -2.341  1.00  0.00
ATOM    767 2HB  MET    49       16.854  -8.306  -3.472  1.00  0.00
ATOM    768 1HG  MET    49       13.893  -8.529  -4.092  1.00  0.00
ATOM    769 2HG  MET    49       14.736  -7.026  -3.650  1.00  0.00
ATOM    770 1HE  MET    49       17.052  -6.360  -6.694  1.00  0.00
ATOM    771 2HE  MET    49       16.349  -5.708  -5.194  1.00  0.00
ATOM    772 3HE  MET    49       17.455  -7.102  -5.127  1.00  0.00
ATOM    773  N   VAL    50       17.126 -11.985  -2.575  1.00  0.00
ATOM    774  CA  VAL    50       18.262 -12.571  -1.870  1.00  0.00
ATOM    775  C   VAL    50       19.549 -12.728  -2.682  1.00  0.00
ATOM    776  O   VAL    50       20.614 -12.254  -2.264  1.00  0.00
ATOM    777  CB  VAL    50       17.925 -13.969  -1.318  1.00  0.00
ATOM    778  CG1 VAL    50       19.198 -14.733  -0.988  1.00  0.00
ATOM    779  CG2 VAL    50       17.039 -13.855  -0.086  1.00  0.00
ATOM    780  H   VAL    50       16.236 -12.463  -2.589  1.00  0.00
ATOM    781  HA  VAL    50       18.584 -11.938  -1.042  1.00  0.00
ATOM    782  HB  VAL    50       17.356 -14.518  -2.068  1.00  0.00
ATOM    783 1HG1 VAL    50       18.941 -15.719  -0.600  1.00  0.00
ATOM    784 2HG1 VAL    50       19.799 -14.845  -1.890  1.00  0.00
ATOM    785 3HG1 VAL    50       19.767 -14.185  -0.237  1.00  0.00
ATOM    786 1HG2 VAL    50       16.809 -14.851   0.291  1.00  0.00
ATOM    787 2HG2 VAL    50       17.560 -13.286   0.684  1.00  0.00
ATOM    788 3HG2 VAL    50       16.112 -13.345  -0.350  1.00  0.00
ATOM    789  N   GLU    51       19.437 -13.345  -3.860  1.00  0.00
ATOM    790  CA  GLU    51       20.594 -13.565  -4.735  1.00  0.00
ATOM    791  C   GLU    51       21.226 -12.262  -5.223  1.00  0.00
ATOM    792  O   GLU    51       22.440 -12.067  -5.093  1.00  0.00
ATOM    793  CB  GLU    51       20.197 -14.415  -5.944  1.00  0.00
ATOM    794  CG  GLU    51       19.920 -15.877  -5.621  1.00  0.00
ATOM    795  CD  GLU    51       19.512 -16.640  -6.851  1.00  0.00
ATOM    796  OE1 GLU    51       19.417 -16.040  -7.895  1.00  0.00
```

ATOM	797	OE2	GLU	51	19.404	-17.841	-6.772	1.00	0.00
ATOM	798	H	GLU	51	18.528	-13.670	-4.158	1.00	0.00
ATOM	799	HA	GLU	51	21.379	-14.086	-4.186	1.00	0.00
ATOM	800	1HB	GLU	51	19.302	-13.962	-6.371	1.00	0.00
ATOM	801	2HB	GLU	51	21.015	-14.351	-6.662	1.00	0.00
ATOM	802	1HG	GLU	51	20.759	-16.382	-5.143	1.00	0.00
ATOM	803	2HG	GLU	51	19.082	-15.832	-4.927	1.00	0.00
ATOM	804	N	TRP	52	20.402	-11.370	-5.768	1.00	0.00
ATOM	805	CA	TRP	52	20.893	-10.094	-6.266	1.00	0.00
ATOM	806	C	TRP	52	21.648	-9.323	-5.181	1.00	0.00
ATOM	807	O	TRP	52	22.726	-8.779	-5.440	1.00	0.00
ATOM	808	CB	TRP	52	19.735	-9.247	-6.797	1.00	0.00
ATOM	809	CG	TRP	52	20.165	-7.921	-7.346	1.00	0.00
ATOM	810	CD1	TRP	52	20.465	-7.635	-8.644	1.00	0.00
ATOM	811	CD2	TRP	52	20.343	-6.702	-6.614	1.00	0.00
ATOM	812	NE1	TRP	52	20.821	-6.315	-8.768	1.00	0.00
ATOM	813	CE2	TRP	52	20.752	-5.720	-7.534	1.00	0.00
ATOM	814	CE3	TRP	52	20.195	-6.347	-5.268	1.00	0.00
ATOM	815	CZ2	TRP	52	21.016	-4.412	-7.156	1.00	0.00
ATOM	816	CZ3	TRP	52	20.458	-5.036	-4.889	1.00	0.00
ATOM	817	CH2	TRP	52	20.856	-4.096	-5.807	1.00	0.00
ATOM	818	H	TRP	52	19.417	-11.583	-5.837	1.00	0.00
ATOM	819	HE1	TRP	52	21.089	-5.856	-9.626	1.00	0.00
ATOM	820	HD1	TRP	52	20.385	-8.452	-9.360	1.00	0.00
ATOM	821	HZ2	TRP	52	21.329	-3.698	-7.917	1.00	0.00
ATOM	822	HH2	TRP	52	21.052	-3.078	-5.469	1.00	0.00
ATOM	823	HZ3	TRP	52	20.340	-4.773	-3.838	1.00	0.00
ATOM	824	HE3	TRP	52	19.880	-7.052	-4.499	1.00	0.00
ATOM	825	HA	TRP	52	21.602	-10.262	-7.077	1.00	0.00
ATOM	826	1HB	TRP	52	19.226	-9.769	-7.607	1.00	0.00
ATOM	827	2HB	TRP	52	19.025	-9.038	-5.997	1.00	0.00
ATOM	828	N	PHE	53	21.115	-9.296	-3.961	1.00	0.00
ATOM	829	CA	PHE	53	21.782	-8.584	-2.877	1.00	0.00
ATOM	830	C	PHE	53	23.091	-9.266	-2.488	1.00	0.00
ATOM	831	O	PHE	53	24.088	-8.593	-2.251	1.00	0.00
ATOM	832	CB	PHE	53	20.862	-8.481	-1.659	1.00	0.00
ATOM	833	CG	PHE	53	21.349	-7.523	-0.610	1.00	0.00
ATOM	834	CD1	PHE	53	21.208	-6.154	-0.782	1.00	0.00
ATOM	835	CD2	PHE	53	21.950	-7.988	0.550	1.00	0.00
ATOM	836	CE1	PHE	53	21.656	-5.271	0.182	1.00	0.00
ATOM	837	CE2	PHE	53	22.398	-7.108	1.516	1.00	0.00
ATOM	838	CZ	PHE	53	22.251	-5.748	1.331	1.00	0.00
ATOM	839	H	PHE	53	20.242	-9.772	-3.785	1.00	0.00
ATOM	840	HD1	PHE	53	20.737	-5.777	-1.690	1.00	0.00
ATOM	841	HE1	PHE	53	22.868	-7.486	2.424	1.00	0.00
ATOM	842	HZ	PHE	53	22.606	-5.053	2.091	1.00	0.00
ATOM	843	HE2	PHE	53	22.868	-7.486	2.424	1.00	0.00
ATOM	844	HD2	PHE	53	22.067	-9.062	0.695	1.00	0.00
ATOM	845	HA	PHE	53	22.043	-7.576	-3.202	1.00	0.00
ATOM	846	1HB	PHE	53	19.875	-8.135	-1.962	1.00	0.00
ATOM	847	2HB	PHE	53	20.772	-9.453	-1.175	1.00	0.00
ATOM	848	N	LEU	54	23.105	-10.592	-2.452	1.00	0.00
ATOM	849	CA	LEU	54	24.320	-11.300	-2.096	1.00	0.00
ATOM	850	C	LEU	54	25.410	-11.043	-3.134	1.00	0.00
ATOM	851	O	LEU	54	26.559	-10.781	-2.775	1.00	0.00
ATOM	852	CB	LEU	54	24.059	-12.806	-1.961	1.00	0.00
ATOM	853	CG	LEU	54	23.082	-13.242	-0.865	1.00	0.00

```
ATOM    854  CD1 LEU    54      22.990 -14.750  -0.689  1.00  0.00
ATOM    855  CD2 LEU    54      23.315 -12.554   0.472  1.00  0.00
ATOM    856  H   LEU    54      22.267 -11.110  -2.673  1.00  0.00
ATOM    857  HA  LEU    54      24.695 -10.925  -1.144  1.00  0.00
ATOM    858 1HB  LEU    54      23.618 -12.998  -2.938  1.00  0.00
ATOM    859 2HB  LEU    54      24.987 -13.369  -1.865  1.00  0.00
ATOM    860  HG  LEU    54      22.101 -12.847  -1.130  1.00  0.00
ATOM    861 1HD1 LEU    54      22.279 -14.982   0.104  1.00  0.00
ATOM    862 2HD1 LEU    54      22.654 -15.205  -1.621  1.00  0.00
ATOM    863 3HD1 LEU    54      23.970 -15.146  -0.424  1.00  0.00
ATOM    864 1HD2 LEU    54      22.584 -12.913   1.198  1.00  0.00
ATOM    865 2HD2 LEU    54      24.321 -12.779   0.827  1.00  0.00
ATOM    866 3HD2 LEU    54      23.206 -11.476   0.351  1.00  0.00
ATOM    867  N   GLU    55      25.038 -11.070  -4.414  1.00  0.00
ATOM    868  CA  GLU    55      25.991 -10.833  -5.502  1.00  0.00
ATOM    869  C   GLU    55      26.614  -9.439  -5.375  1.00  0.00
ATOM    870  O   GLU    55      27.831  -9.266  -5.534  1.00  0.00
ATOM    871  CB  GLU    55      25.304 -10.991  -6.860  1.00  0.00
ATOM    872  CG  GLU    55      24.914 -12.421  -7.205  1.00  0.00
ATOM    873  CD  GLU    55      24.222 -12.492  -8.538  1.00  0.00
ATOM    874  OE1 GLU    55      24.027 -11.464  -9.141  1.00  0.00
ATOM    875  OE2 GLU    55      23.989 -13.581  -9.007  1.00  0.00
ATOM    876  H   GLU    55      24.072 -11.259  -4.640  1.00  0.00
ATOM    877  HA  GLU    55      26.809 -11.552  -5.440  1.00  0.00
ATOM    878 1HB  GLU    55      24.410 -10.367  -6.839  1.00  0.00
ATOM    879 2HB  GLU    55      25.995 -10.612  -7.613  1.00  0.00
ATOM    880 1HG  GLU    55      25.755 -13.114  -7.196  1.00  0.00
ATOM    881 2HG  GLU    55      24.213 -12.692  -6.417  1.00  0.00
ATOM    882  N   MET    56      25.769  -8.451  -5.089  1.00  0.00
ATOM    883  CA  MET    56      26.216  -7.077  -4.935  1.00  0.00
ATOM    884  C   MET    56      27.186  -6.983  -3.760  1.00  0.00
ATOM    885  O   MET    56      28.213  -6.302  -3.842  1.00  0.00
ATOM    886  CB  MET    56      25.018  -6.152  -4.734  1.00  0.00
ATOM    887  CG  MET    56      24.160  -5.953  -5.976  1.00  0.00
ATOM    888  SD  MET    56      24.925  -4.849  -7.180  1.00  0.00
ATOM    889  CE  MET    56      24.828  -3.285  -6.314  1.00  0.00
ATOM    890  H   MET    56      24.788  -8.663  -4.976  1.00  0.00
ATOM    891  HA  MET    56      26.756  -6.760  -5.827  1.00  0.00
ATOM    892 1HB  MET    56      24.412  -6.586  -3.940  1.00  0.00
ATOM    893 2HB  MET    56      25.412  -5.189  -4.407  1.00  0.00
ATOM    894 1HG  MET    56      23.997  -6.927  -6.436  1.00  0.00
ATOM    895 2HG  MET    56      23.204  -5.534  -5.664  1.00  0.00
ATOM    896 1HE  MET    56      25.263  -2.499  -6.932  1.00  0.00
ATOM    897 2HE  MET    56      23.784  -3.048  -6.106  1.00  0.00
ATOM    898 3HE  MET    56      25.378  -3.355  -5.375  1.00  0.00
ATOM    899  N   LEU    57      26.852  -7.654  -2.665  1.00  0.00
ATOM    900  CA  LEU    57      27.698  -7.642  -1.481  1.00  0.00
ATOM    901  C   LEU    57      29.087  -8.163  -1.821  1.00  0.00
ATOM    902  O   LEU    57      30.090  -7.487  -1.588  1.00  0.00
ATOM    903  CB  LEU    57      27.076  -8.476  -0.353  1.00  0.00
ATOM    904  CG  LEU    57      25.889  -7.857   0.389  1.00  0.00
ATOM    905  CD1 LEU    57      25.260  -8.780   1.422  1.00  0.00
ATOM    906  CD2 LEU    57      26.188  -6.493   0.995  1.00  0.00
ATOM    907  H   LEU    57      25.992  -8.184  -2.653  1.00  0.00
ATOM    908  HA  LEU    57      27.820  -6.618  -1.130  1.00  0.00
ATOM    909 1HB  LEU    57      26.735  -9.332  -0.933  1.00  0.00
ATOM    910 2HB  LEU    57      27.825  -8.801   0.369  1.00  0.00
```

```
ATOM    911  HG  LEU    57      25.143  -7.589  -0.360  1.00  0.00
ATOM    912 1HD1 LEU    57      24.426  -8.271   1.905  1.00  0.00
ATOM    913 2HD1 LEU    57      24.897  -9.683   0.930  1.00  0.00
ATOM    914 3HD1 LEU    57      26.004  -9.048   2.171  1.00  0.00
ATOM    915 1HD2 LEU    57      25.299  -6.119   1.503  1.00  0.00
ATOM    916 2HD2 LEU    57      27.005  -6.584   1.711  1.00  0.00
ATOM    917 3HD2 LEU    57      26.473  -5.798   0.205  1.00  0.00
ATOM    918  N   LYS    58      29.133  -9.359  -2.400  1.00  0.00
ATOM    919  CA  LYS    58      30.393  -9.984  -2.783  1.00  0.00
ATOM    920  C   LYS    58      31.152  -9.139  -3.801  1.00  0.00
ATOM    921  O   LYS    58      32.365  -8.974  -3.686  1.00  0.00
ATOM    922  CB  LYS    58      30.147 -11.384  -3.349  1.00  0.00
ATOM    923  CG  LYS    58      29.751 -12.422  -2.307  1.00  0.00
ATOM    924  CD  LYS    58      29.540 -13.789  -2.941  1.00  0.00
ATOM    925  CE  LYS    58      29.149 -14.828  -1.901  1.00  0.00
ATOM    926  NZ  LYS    58      28.917 -16.166  -2.511  1.00  0.00
ATOM    927  H   LYS    58      28.269  -9.849  -2.580  1.00  0.00
ATOM    928 1HZ  LYS    58      28.660 -16.824  -1.789  1.00  0.00
ATOM    929 2HZ  LYS    58      28.170 -16.102  -3.189  1.00  0.00
ATOM    930 3HZ  LYS    58      29.761 -16.479  -2.967  1.00  0.00
ATOM    931  HA  LYS    58      31.042 -10.071  -1.911  1.00  0.00
ATOM    932 1HB  LYS    58      29.353 -11.293  -4.091  1.00  0.00
ATOM    933 2HB  LYS    58      31.068 -11.697  -3.840  1.00  0.00
ATOM    934 1HG  LYS    58      30.545 -12.485  -1.562  1.00  0.00
ATOM    935 2HG  LYS    58      28.828 -12.097  -1.827  1.00  0.00
ATOM    936 1HD  LYS    58      28.750 -13.707  -3.689  1.00  0.00
ATOM    937 2HD  LYS    58      30.467 -14.094  -3.426  1.00  0.00
ATOM    938 1HE  LYS    58      29.952 -14.897  -1.169  1.00  0.00
ATOM    939 2HE  LYS    58      28.237 -14.491  -1.408  1.00  0.00
ATOM    940  N   ALA    59      30.432  -8.562  -4.762  1.00  0.00
ATOM    941  CA  ALA    59      31.063  -7.732  -5.782  1.00  0.00
ATOM    942  C   ALA    59      31.773  -6.526  -5.161  1.00  0.00
ATOM    943  O   ALA    59      32.662  -5.943  -5.781  1.00  0.00
ATOM    944  CB  ALA    59      30.033  -7.273  -6.803  1.00  0.00
ATOM    945  H   ALA    59      29.432  -8.702  -4.785  1.00  0.00
ATOM    946  HA  ALA    59      31.821  -8.324  -6.295  1.00  0.00
ATOM    947 1HB  ALA    59      30.520  -6.655  -7.557  1.00  0.00
ATOM    948 2HB  ALA    59      29.583  -8.142  -7.282  1.00  0.00
ATOM    949 3HB  ALA    59      29.259  -6.692  -6.303  1.00  0.00
ATOM    950  N   LYS    60      31.343  -6.118  -3.968  1.00  0.00
ATOM    951  CA  LYS    60      31.959  -4.978  -3.287  1.00  0.00
ATOM    952  C   LYS    60      32.936  -5.508  -2.233  1.00  0.00
ATOM    953  O   LYS    60      33.579  -4.737  -1.509  1.00  0.00
ATOM    954  CB  LYS    60      30.887  -4.086  -2.659  1.00  0.00
ATOM    955  CG  LYS    60      30.206  -3.138  -3.637  1.00  0.00
ATOM    956  CD  LYS    60      31.161  -2.053  -4.112  1.00  0.00
ATOM    957  CE  LYS    60      30.466  -1.072  -5.045  1.00  0.00
ATOM    958  NZ  LYS    60      31.400  -0.031  -5.554  1.00  0.00
ATOM    959  H   LYS    60      30.577  -6.605  -3.525  1.00  0.00
ATOM    960 1HZ  LYS    60      30.901   0.598  -6.168  1.00  0.00
ATOM    961 2HZ  LYS    60      31.781   0.489  -4.776  1.00  0.00
ATOM    962 3HZ  LYS    60      32.151  -0.473  -6.064  1.00  0.00
ATOM    963  HA  LYS    60      32.530  -4.385  -4.003  1.00  0.00
ATOM    964 1HB  LYS    60      30.142  -4.747  -2.215  1.00  0.00
ATOM    965 2HB  LYS    60      31.372  -3.508  -1.872  1.00  0.00
ATOM    966 1HG  LYS    60      29.857  -3.716  -4.493  1.00  0.00
ATOM    967 2HG  LYS    60      29.353  -2.679  -3.138  1.00  0.00
```

```
ATOM    968 1HD  LYS    60       31.541  -1.519  -3.240  1.00  0.00
ATOM    969 2HD  LYS    60       31.991  -2.526  -4.637  1.00  0.00
ATOM    970 1HE  LYS    60       30.054  -1.631  -5.884  1.00  0.00
ATOM    971 2HE  LYS    60       29.655  -0.594  -4.495  1.00  0.00
ATOM    972  N   GLY    61       33.033  -6.831  -2.150  1.00  0.00
ATOM    973  CA  GLY    61       33.918  -7.463  -1.190  1.00  0.00
ATOM    974  C   GLY    61       33.420  -7.265   0.232  1.00  0.00
ATOM    975  O   GLY    61       34.210  -7.261   1.180  1.00  0.00
ATOM    976  H   GLY    61       32.480  -7.408  -2.768  1.00  0.00
ATOM    977 1HA  GLY    61       33.969  -8.531  -1.402  1.00  0.00
ATOM    978 2HA  GLY    61       34.912  -7.027  -1.281  1.00  0.00
ATOM    979  N   ILE    62       32.111  -7.077   0.387  1.00  0.00
ATOM    980  CA  ILE    62       31.538  -6.877   1.705  1.00  0.00
ATOM    981  C   ILE    62       31.237  -8.230   2.302  1.00  0.00
ATOM    982  O   ILE    62       30.619  -9.082   1.661  1.00  0.00
ATOM    983  CB  ILE    62       30.264  -6.015   1.642  1.00  0.00
ATOM    984  CG1 ILE    62       30.588  -4.622   1.096  1.00  0.00
ATOM    985  CG2 ILE    62       29.622  -5.914   3.017  1.00  0.00
ATOM    986  CD1 ILE    62       29.366  -3.790   0.781  1.00  0.00
ATOM    987  H   ILE    62       31.509  -7.074  -0.424  1.00  0.00
ATOM    988  HA  ILE    62       32.261  -6.407   2.371  1.00  0.00
ATOM    989  HB  ILE    62       29.562  -6.470   0.944  1.00  0.00
ATOM    990 1HG2 ILE    62       28.723  -5.301   2.955  1.00  0.00
ATOM    991 2HG2 ILE    62       29.358  -6.910   3.369  1.00  0.00
ATOM    992 3HG2 ILE    62       30.325  -5.457   3.714  1.00  0.00
ATOM    993 1HG1 ILE    62       31.191  -4.112   1.846  1.00  0.00
ATOM    994 2HG1 ILE    62       31.177  -4.759   0.189  1.00  0.00
ATOM    995 1HD1 ILE    62       29.676  -2.817   0.399  1.00  0.00
ATOM    996 2HD1 ILE    62       28.762  -4.299   0.029  1.00  0.00
ATOM    997 3HD1 ILE    62       28.776  -3.651   1.686  1.00  0.00
ATOM    998  N   PRO    63       31.766  -8.438   3.504  1.00  0.00
ATOM    999  CA  PRO    63       31.593  -9.682   4.241  1.00  0.00
ATOM   1000  C   PRO    63       30.208  -9.764   4.882  1.00  0.00
ATOM   1001  O   PRO    63       29.697  -8.763   5.395  1.00  0.00
ATOM   1002  CB  PRO    63       32.659  -9.772   5.320  1.00  0.00
ATOM   1003  CG  PRO    63       33.164  -8.363   5.491  1.00  0.00
ATOM   1004  CD  PRO    63       32.903  -7.674   4.031  1.00  0.00
ATOM   1005 1HD  PRO    63       32.576  -6.780   4.089  1.00  0.00
ATOM   1006 2HD  PRO    63       33.592  -7.854   3.399  1.00  0.00
ATOM   1007 1HG  PRO    63       32.611  -7.936   5.970  1.00  0.00
ATOM   1008 2HG  PRO    63       34.101  -8.398   5.492  1.00  0.00
ATOM   1009 1HB  PRO    63       32.144  -9.957   6.053  1.00  0.00
ATOM   1010 2HB  PRO    63       33.274 -10.223   4.910  1.00  0.00
ATOM   1011  HA  PRO    63       31.700 -10.461   3.629  1.00  0.00
ATOM   1012  N   PHE    64       29.608 -10.950   4.847  1.00  0.00
ATOM   1013  CA  PHE    64       28.281 -11.156   5.423  1.00  0.00
ATOM   1014  C   PHE    64       28.078 -12.628   5.755  1.00  0.00
ATOM   1015  O   PHE    64       28.870 -13.482   5.353  1.00  0.00
ATOM   1016  CB  PHE    64       27.198 -10.664   4.461  1.00  0.00
ATOM   1017  CG  PHE    64       27.174 -11.397   3.150  1.00  0.00
ATOM   1018  CD1 PHE    64       26.382 -12.524   2.984  1.00  0.00
ATOM   1019  CD2 PHE    64       27.943 -10.963   2.081  1.00  0.00
ATOM   1020  CE1 PHE    64       26.359 -13.199   1.778  1.00  0.00
ATOM   1021  CE2 PHE    64       27.921 -11.636   0.874  1.00  0.00
ATOM   1022  CZ  PHE    64       27.128 -12.756   0.724  1.00  0.00
ATOM   1023  H   PHE    64       30.082 -11.728   4.411  1.00  0.00
ATOM   1024  HD1 PHE    64       25.773 -12.874   3.817  1.00  0.00
```

```
ATOM   1025  HE1 PHE    64      28.530 -11.284   0.041  1.00  0.00
ATOM   1026  HZ  PHE    64      27.111 -13.288  -0.226  1.00  0.00
ATOM   1027  HE2 PHE    64      28.530 -11.284   0.041  1.00  0.00
ATOM   1028  HD2 PHE    64      28.570 -10.079   2.200  1.00  0.00
ATOM   1029  HA  PHE    64      28.193 -10.602   6.359  1.00  0.00
ATOM   1030 1HB  PHE    64      26.213 -10.792   4.908  1.00  0.00
ATOM   1031 2HB  PHE    64      27.356  -9.612   4.227  1.00  0.00
ATOM   1032  N   THR    65      27.010 -12.908   6.490  1.00  0.00
ATOM   1033  CA  THR    65      26.657 -14.264   6.876  1.00  0.00
ATOM   1034  C   THR    65      25.144 -14.312   7.083  1.00  0.00
ATOM   1035  O   THR    65      24.502 -13.270   7.253  1.00  0.00
ATOM   1036  CB  THR    65      27.400 -14.700   8.152  1.00  0.00
ATOM   1037  OG1 THR    65      27.175 -16.096   8.386  1.00  0.00
ATOM   1038  CG2 THR    65      26.912 -13.904   9.352  1.00  0.00
ATOM   1039  H   THR    65      26.418 -12.148   6.794  1.00  0.00
ATOM   1040  HG1 THR    65      27.639 -16.365   9.182  1.00  0.00
ATOM   1041  HA  THR    65      26.910 -14.954   6.071  1.00  0.00
ATOM   1042  HB  THR    65      28.468 -14.531   8.015  1.00  0.00
ATOM   1043 1HG2 THR    65      27.449 -14.226  10.244  1.00  0.00
ATOM   1044 2HG2 THR    65      27.093 -12.843   9.182  1.00  0.00
ATOM   1045 3HG2 THR    65      25.845 -14.073   9.490  1.00  0.00
ATOM   1046  N   VAL    66      24.568 -15.502   6.968  1.00  0.00
ATOM   1047  CA  VAL    66      23.133 -15.676   7.139  1.00  0.00
ATOM   1048  C   VAL    66      22.850 -15.875   8.619  1.00  0.00
ATOM   1049  O   VAL    66      23.307 -16.850   9.221  1.00  0.00
ATOM   1050  CB  VAL    66      22.607 -16.869   6.319  1.00  0.00
ATOM   1051  CG1 VAL    66      21.113 -17.051   6.542  1.00  0.00
ATOM   1052  CG2 VAL    66      22.903 -16.672   4.841  1.00  0.00
ATOM   1053  H   VAL    66      25.140 -16.307   6.756  1.00  0.00
ATOM   1054  HA  VAL    66      22.587 -14.781   6.840  1.00  0.00
ATOM   1055  HB  VAL    66      23.133 -17.772   6.629  1.00  0.00
ATOM   1056 1HG1 VAL    66      20.758 -17.899   5.955  1.00  0.00
ATOM   1057 2HG1 VAL    66      20.923 -17.237   7.599  1.00  0.00
ATOM   1058 3HG1 VAL    66      20.586 -16.149   6.231  1.00  0.00
ATOM   1059 1HG2 VAL    66      22.525 -17.524   4.277  1.00  0.00
ATOM   1060 2HG2 VAL    66      22.417 -15.761   4.492  1.00  0.00
ATOM   1061 3HG2 VAL    66      23.980 -16.589   4.693  1.00  0.00
ATOM   1062  N   TYR    67      22.158 -14.909   9.207  1.00  0.00
ATOM   1063  CA  TYR    67      21.819 -14.977  10.616  1.00  0.00
ATOM   1064  C   TYR    67      20.524 -15.780  10.770  1.00  0.00
ATOM   1065  O   TYR    67      20.451 -16.700  11.586  1.00  0.00
ATOM   1066  CB  TYR    67      21.677 -13.570  11.201  1.00  0.00
ATOM   1067  CG  TYR    67      21.368 -13.550  12.682  1.00  0.00
ATOM   1068  CD1 TYR    67      22.296 -13.998  13.610  1.00  0.00
ATOM   1069  CD2 TYR    67      20.149 -13.081  13.147  1.00  0.00
ATOM   1070  CE1 TYR    67      22.019 -13.982  14.963  1.00  0.00
ATOM   1071  CE2 TYR    67      19.860 -13.060  14.498  1.00  0.00
ATOM   1072  CZ  TYR    67      20.799 -13.511  15.403  1.00  0.00
ATOM   1073  OH  TYR    67      20.517 -13.492  16.750  1.00  0.00
ATOM   1074  H   TYR    67      21.861 -14.111   8.664  1.00  0.00
ATOM   1075  HH  TYR    67      21.238 -13.827  17.290  1.00  0.00
ATOM   1076  HD1 TYR    67      23.258 -14.368  13.255  1.00  0.00
ATOM   1077  HE1 TYR    67      22.758 -14.338  15.681  1.00  0.00
ATOM   1078  HE2 TYR    67      18.895 -12.688  14.843  1.00  0.00
ATOM   1079  HD2 TYR    67      19.411 -12.726  12.426  1.00  0.00
ATOM   1080  HA  TYR    67      22.604 -15.503  11.161  1.00  0.00
ATOM   1081 1HB  TYR    67      22.618 -13.048  11.019  1.00  0.00
```

```
ATOM   1082 2HB  TYR    67      20.875 -13.074  10.656  1.00  0.00
ATOM   1083  N   VAL    68      19.508 -15.443   9.976  1.00  0.00
ATOM   1084  CA  VAL    68      18.226 -16.143  10.024  1.00  0.00
ATOM   1085  C   VAL    68      17.973 -16.655   8.606  1.00  0.00
ATOM   1086  O   VAL    68      17.898 -15.867   7.661  1.00  0.00
ATOM   1087  CB  VAL    68      17.095 -15.218  10.511  1.00  0.00
ATOM   1088  CG1 VAL    68      15.767 -15.960  10.519  1.00  0.00
ATOM   1089  CG2 VAL    68      17.409 -14.676  11.897  1.00  0.00
ATOM   1090  H   VAL    68      19.627 -14.682   9.323  1.00  0.00
ATOM   1091  HA  VAL    68      18.273 -17.012  10.682  1.00  0.00
ATOM   1092  HB  VAL    68      17.028 -14.359   9.843  1.00  0.00
ATOM   1093 1HG1 VAL    68      14.979 -15.291  10.866  1.00  0.00
ATOM   1094 2HG1 VAL    68      15.535 -16.302   9.510  1.00  0.00
ATOM   1095 3HG1 VAL    68      15.834 -16.818  11.188  1.00  0.00
ATOM   1096 1HG2 VAL    68      16.600 -14.024  12.225  1.00  0.00
ATOM   1097 2HG2 VAL    68      17.513 -15.506  12.596  1.00  0.00
ATOM   1098 3HG2 VAL    68      18.340 -14.110  11.864  1.00  0.00
ATOM   1099  N   GLU    69      17.876 -17.975   8.474  1.00  0.00
ATOM   1100  CA  GLU    69      17.643 -18.661   7.194  1.00  0.00
ATOM   1101  C   GLU    69      16.306 -18.385   6.520  1.00  0.00
ATOM   1102  O   GLU    69      16.242 -18.199   5.304  1.00  0.00
ATOM   1103  CB  GLU    69      17.763 -20.177   7.369  1.00  0.00
ATOM   1104  CG  GLU    69      19.184 -20.673   7.597  1.00  0.00
ATOM   1105  CD  GLU    69      19.218 -22.164   7.785  1.00  0.00
ATOM   1106  OE1 GLU    69      18.173 -22.769   7.774  1.00  0.00
ATOM   1107  OE2 GLU    69      20.294 -22.712   7.829  1.00  0.00
ATOM   1108  H   GLU    69      17.968 -18.542   9.305  1.00  0.00
ATOM   1109  HA  GLU    69      18.381 -18.336   6.460  1.00  0.00
ATOM   1110 1HB  GLU    69      17.143 -20.449   8.223  1.00  0.00
ATOM   1111 2HB  GLU    69      17.359 -20.635   6.466  1.00  0.00
ATOM   1112 1HG  GLU    69      19.874 -20.392   6.802  1.00  0.00
ATOM   1113 2HG  GLU    69      19.481 -20.183   8.524  1.00  0.00
ATOM   1114  N   GLU    70      15.238 -18.438   7.308  1.00  0.00
ATOM   1115  CA  GLU    70      13.899 -18.211   6.806  1.00  0.00
ATOM   1116  C   GLU    70      13.075 -17.559   7.908  1.00  0.00
ATOM   1117  O   GLU    70      12.766 -18.175   8.934  1.00  0.00
ATOM   1118  CB  GLU    70      13.272 -19.526   6.338  1.00  0.00
ATOM   1119  CG  GLU    70      11.910 -19.373   5.676  1.00  0.00
ATOM   1120  CD  GLU    70      11.367 -20.702   5.229  1.00  0.00
ATOM   1121  OE1 GLU    70      12.031 -21.691   5.430  1.00  0.00
ATOM   1122  OE2 GLU    70      10.243 -20.744   4.790  1.00  0.00
ATOM   1123  H   GLU    70      15.364 -18.643   8.289  1.00  0.00
ATOM   1124  HA  GLU    70      13.933 -17.522   5.962  1.00  0.00
ATOM   1125 1HB  GLU    70      13.969 -19.978   5.632  1.00  0.00
ATOM   1126 2HB  GLU    70      13.179 -20.165   7.216  1.00  0.00
ATOM   1127 1HG  GLU    70      11.174 -18.875   6.307  1.00  0.00
ATOM   1128 2HG  GLU    70      12.117 -18.754   4.804  1.00  0.00
TER
END
```
